# Overexpression of TECPR1 improved cognitive function of P301S‐tau mice via activation of autophagy in the early and late process

**DOI:** 10.1111/acel.14404

**Published:** 2024-11-07

**Authors:** Ting Li, Ruijuan Liu, Ye He, Bingge Zhang, Xuexiang Rui, Xifei Yang, Jian‐Zhi Wang, Juan Zeng, Gang Li, Xiao Li, Gong‐Ping Liu

**Affiliations:** ^1^ Department of Pathology Renmin Hospital of Wuhan University Wuhan China; ^2^ Department of Pathophysiology, School of Basic Medicine and the Collaborative Innovation Center for Brain Science, Key Laboratory of Ministry of Education of China and Hubei Province for Neurological Disorders, Tongji Medical College Huazhong University of Science and Technology Wuhan China; ^3^ Key Laboratory of Modern Toxicology of Shenzhen, Shenzhen Medical Key Subject of Modern Toxicology Shenzhen Center for Disease Control and Prevention Shenzhen China; ^4^ Department of Neurology, Union Hospital, Tongji Medical College Huazhong University of Science and Technology Wuhan China; ^5^ Department of Pathology Wuhan No. 1 Hospital Wuhan China; ^6^ Co‐Innovation Center of Neuroregeneration Nantong University Nantong Jiangsu China

**Keywords:** autophagy, cognitive function, synapse, tau, TECPR1

## Abstract

Autophagy disorders in AD patients and animal models were well known, however, the effect of P301S‐tau on autophagy is not clear. Here, we found that autophagy related protein Tectonin Beta‐Propeller Repeat‐Containing Protein 1 (TECPR1) decreased in the hippocampus of P301S‐tau transgenic mice by proteomics, which was proved in vivo and in vitro, and P301S‐tau induced autophagic deficits in early and late process. TECPR1 overexpression attenuated P301S‐tau induced autophagy defects via promoting autophagosome generation and autophagosome and lysosomes fusion. We also found that TECPR1 overexpression ameliorated the behavior disorders of P301S‐tau mice with promoting tau degradation, improving synaptic plasticity and neuron loss. Lastly, CQ or 3‐MA treatment reversed TECPR1 induced improvement effects on autophagic and cognitive disorders, further proved that, TECPR1 activated the early and late process of autophagy to ameliorate the cognition of P301S‐tau mice. Our data suggest that TECPR1 is a potential therapy target for AD.

## Graphical abstract

P301S‐tau induced autophagic defects in the early and late process by inhibiting the expression of TECPR1, which enrich the autophagy research in tauopathies. Overexpression of TECPR1 can activate autophagy promote autophagosome generation and fusion with lysosomes, thus accelerate tau degradation, thereby improving synaptic plasticity and cognitive function in P301S‐tau mice.

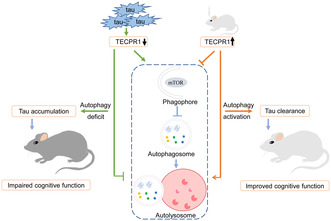

**Abbreviations**
3‐MA: 3‐Methyladenine
AAV: adeno‐associated virus
AD: alzheimer's disease
ATG12: autophagy related gene 12
ATG13: autophagy related gene 13
ATG16L1: autophagy related gene 16L1
ATG5: autophagy related gene 5
ATP6V0D1: V‐type proton ATPase subunit d 1
ATP6V0D2: V‐type proton ATPase subunit d 2
Baf A1: bafilomycin A1
CQ: chloroquine
FIP200: focal adhesion kinase family interacting protein of 200 kDa
GFAP: glial fibrillary acidic protein
GluNR1: N‐methyl‐D‐aspartate receptor 1
HT7: human tau
IBA1: allograft inflammatory factor 1
ISG: interferon‐stimulating gene
LAMP1: lysosome‐associated membrane protein 2
LAMP2: lysosome‐associated membrane protein 2
LAPTM4a: lysosomal‐associated transmembrane protein 4A
LC3: microtubule associated protein 1 light chain 3
mTORC1: mechanistic target of rapamycin complex 1
P62: sequestosome 1
PSD93: postsynaptic dense‐93
PSD95: postsynaptic dense‐95
SYP: synaptophysin
SYT: synaptotagmin
TECPR1: tectonin beta‐propeller repeat‐containing protein 1
ULK1: Unc‐51‐like kinase 1
VPS15: vacuolar Protein Sorting 15
VPS34: vacuolar Protein Sorting 34
WT: wild‐type

## 1 INTRODUCTION

Microtubule associated protein tau mainly present in neurons, most abundant in the cytoplasm of axons, and also partially present in the presynaptic and postsynaptic membranes, and related to the nuclear membrane (Knopman et al., 2021; Li & Gotz, 2017). The main function of tau is to promote the assembly of microtubules and stabilize them by binding to microtubules, which is essential for axonal transport in normal neurons (Wang & Liu, 2008). Tau can interact with various structural and functional proteins, playing a crucial role not only in maintaining normal microtubule structure, but also in neuronal signal transduction (Wang & Liu, 2008), and can also participate in regulating cell activity through phosphorylation (Bandyopadhyay et al., 2007). However, in pathological conditions, tau can be modified by various post‐translational modifications, causing damage to brain function and ultimately leading to neurodegeneration (Li & Gotz, 2017). The post‐translational modifications of tau include phosphorylation, glycosylation, ISG, hematoxylinization, nitration, ubiquitination, and truncation (Wang & Liu, 2008). Tau phosphorylation is the most extensively studied, as tau phosphorylation levels in brains of neurodegenerative patients, including AD and frontotemporal dementia, are significantly elevated (Khatoon et al., 1992). Neurofibrillary tangles formed by excessive phosphorylation of tau within neurons are one of the typical pathological features of AD (Braak & Braak, 1991; Therriault et al., 2022), which are closely related to the occurrence of neurodegeneration and clinical symptoms in space and time (Ossenkoppele et al., 2022). Postmortem autopsy suggests that tau fiber tangles is closely related to cognitive decline (Nelson et al., 2012). In vitro studies have shown that tau lesions can induce synaptic loss and reduce neural network function (Spires‐Jones & Hyman, 2014). Neuroimaging studies have shown that the time and location of tau lesions correspond to the onset and type of cognitive impairment (Scholl et al., 2016).

Autophagy is a highly conserved biological process that maintains cellular homeostasis. Through autophagy, substances to be degraded is encapsulated in autophagosomes and transported to lysosomes for degradation, thereby clearing misfolded proteins and damaged organelles to maintain normal cellular function (Zhang et al., 2021). Particularly, autophagy plays an indispensable role in clearing abnormally accumulated proteins in neurons (Chung et al., 2019). Research has shown that there are autophagic disorders including the formation and degradation of autophagosomes in the brain of AD patients (Zhang et al., 2022). Autophagy‐lysosomal system dysfunction can lead to the formation of tau oligomers and the aggregation of insoluble tau (Li et al., 2017). Moreover, studies on samples from AD patients and elderly individuals who died of natural aging have shown a correlation between hyperphosphorylated tau and autophagy biomarker microtubule associated protein 1 light chain 3 (LC3) and autophagy substrate protein ubiquitin binding protein 1 (Sequestosome 1, p62) positive autophagosomes in AD patient samples (Piras et al., 2016; Uddin et al., 2019). In AD patients' brain, the autophagy process is blocked, and the degradation of autophagosomes is hindered, leading to tau accumulation in neurons, resulting in impaired tau clearance, intensified tau accumulation, and accelerated learning and memory impairments (Feng et al., 2020). These findings demonstrate that autophagic impairments may be one major reason for AD.

Tectonin Beta‐Propeller Repeat‐Containing Protein 1 (TECPR1) is a highly conserved protein that contains β—propeller repeat domains, have two WD repeat domains (TR), two dysferlin domains, an unstructured region, and 1 PH domain (Ogawa et al., 2011). TECPR1 is widely expressed in systemic organs and at high levels in the brain. TECPR1 locates on the lysosome membrane and also serves as a binding factor between autophagosomes and lysosomes. By recruiting ATG5‐ATG12‐ATG16L1 complexes on the autophagosome membrane, TECPR1 promotes autophagosomes and lysosomes fusion, accelerating the degradation process of autophagy (Kim et al., 2015). Moreover, TECPR1 can also directly bind with LC3 to accelerate the autophagic degradation processes (Wetzel et al., 2020). In addition, studies have also found that TECPR1 can repair damaged lysosomes (Corkery et al., 2023). On the damaged membrane, TECPR1 recruits ATG5‐ATG12 complexes to the damaged lysosomes and catalyzes LC3 lipolysis to form alternative E3 like binding complexes, promoting lysosomal recovery (Corkery et al., 2023). Previous studies have found that TECPR1 can promote the clearance of protein aggregates in neural stem cells (Wetzel et al., 2020). However, the role of TECPR1 in tauopathy has not been reported yet.

In this study, we found that, TECPR1 protein level significantly decreased in the hippocampus of P301S‐tau transgenic mice. Overexpression of TECPR1 reversed the cognitive deficits of P301S‐tau mice via activation of autophagy in both early and late process. The results suggest that TECPR1 may be a potential new target for treating AD.

## 2 RESULTS

### 2.1 P301S‐tau induces autophagy defects and downregulates TECPR1 protein level

**FIGURE 1:**
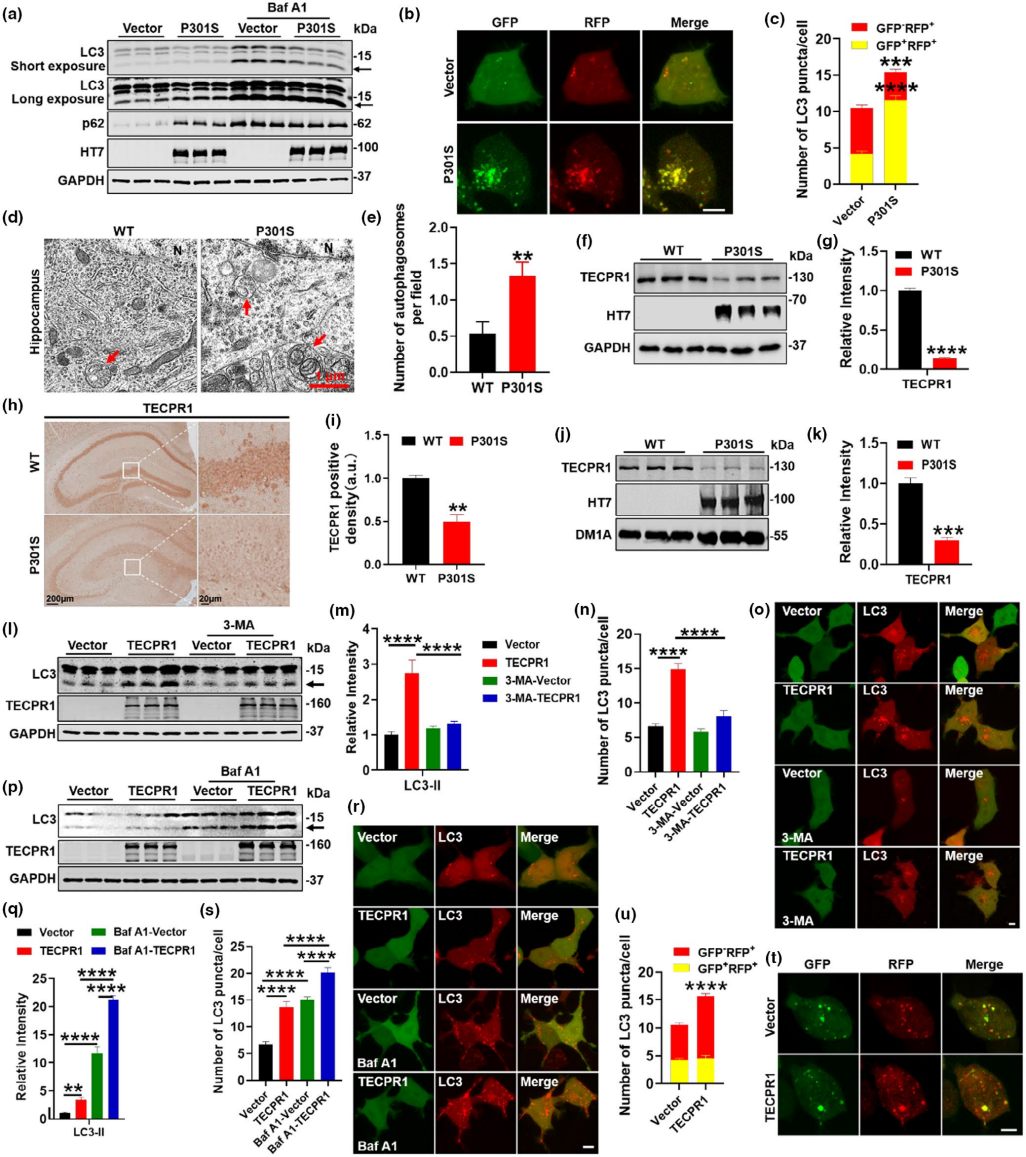
P301S tau induced autophagy defects and downregulated TECPR1 protein level, which can activate autophagy. (a) HEK293 cells were transfected with GFP‐P301S‐tau or its vector for 24 h and then treated with Baf A1 (100 nM) for 24 h, LC3‐II and p62 proteins were detected by western blotting. The black arrow indicates LC3‐II. *N* = 3 each group. (b, c) HEK293 cells were transfected with 3 × FLAG‐P301S‐tau or its vector and GFP‐RFP‐LC3 for 24 h, LC3 puncta of live cells were detected (b) and quantitative analysis (c). Scale bar: 5 μm. At least 25 cells were analyzed for each group. (d, e) The number of autophagosomes in P301S‐tau mice was significantly increased compared with WT mice measured by transmission electron microscopy (d) and quantitative analysis (e). Red arrow, autophagosome; N, nucleus; *N* = 3 mice/group. (f–i) The TECPR1 protein level of hippocampus in 9‐month‐old P301S‐tau mice was detected and quantified by western blotting (f, g) and immunohistochemistry (h, i). *N* = 3 each group. (j, k) AAV‐P301S‐tau virus was injected into hippocampus of 2‐month‐old C57 mice. The TECPR1 protein level of the hippocampus was detected by western blotting (j) and quantitative analysis (k). *N* = 3 each group. Scale bar: 5 μm. At least 25 cells each group. (l, m) HEK293 cells were transfected with CFP‐TECPR1 or its vector for 24 h and then treated with 3‐MA (5 mM) for 24 h, LC3‐II protein was detected by western blotting (l) and quantitative analysis (m). *N* = 3 each group. (n, o) HEK293 cells were transfected with GFP‐TECPR1 or its vector and mCherry‐LC3 for 24 h and then treated with 3‐MA (5 mM) for 24 h, LC3 puncta of live cells were detected (o) and quantitative analysis (n). Scale bar: 5 μm. At least 25 cells were analyzed for each group. (p, q) HEK293 cells were transfected with CFP‐TECPR1 or its vector for 24 h and then treated with Baf A1 (100 nM) for 24 h, LC3‐II protein was detected by western blotting (p) and quantitative analysis (q). *N* = 3 each group. (r, s) HEK293 cells were transfected with HA‐TECPR1 or its vector and mCherry‐LC3 for 24 h and then treated with Baf A1 (100 nM) for 24 h, LC3 puncta of live cells were detected (r) and quantitative analysis (s). Scale bar: 5 μm. At least 25 cells were analyzed for each group. (t, u) HEK293 cells were transfected with HA‐TECPR1 or its vector and GFP‐RFP‐LC3 for 48 h, LC3 puncta of live cells were detected (t) and quantitative analysis (u). Scale bar: 5 μm. At least 25 cells were analyzed for each group. All data were presented as mean ± SEM. one‐way ANOVA test followed by Tukey's post hoc test for m, n, q, s, Unpaired *t*‐test for others. **p < 0.01, ***p < 0.001, ****p < 0.0001 versus WT or Vector.

Accumulated increasing evidences have indicated that tauopathies induced learning and memory impairments are also intimately associated with autophagy disorders (Tumurbaatar et al., 2023; Zhang et al., 2021). To further investigate the effect of P301S‐tau on autophagy, we first transiently transfected P301S‐tau in HEK293 cells and treated with Baf A1 to inhibit autophagosomes and lysosomes fusion. We observed that after overexpression of P301S‐tau, LC3‐II (long exposure) and p62 protein levels were significantly elevated, indicating that P301S‐tau may lead to impaired autophagy. After Baf A1 treatment, LC3‐II proteins were significantly increased in both Vector and P301S groups, however, LC3‐II proteins in the P301S group (short exposure) were significantly lower than in the Vector, indicating that P301S‐tau lead to obstacles in autophagosome formation (Figure 1a, Figure S1).

In order to investigate the effect of P301S‐tau on the fusion of autophagosomes and lysosomes, GFP‐RFP‐LC3 and 3 × FLAG‐P301S‐tau or empty plasmids were co‐transfected into HEK293 cells. If the autophagosome entered the lysosomes, acid‐sensitive GFP fluorescence would be quenched. Therefore, the effect of P301S‐tau on late stage autophagosomes was evaluated by detecting the number of yellow fluorescent puncta (both GFP and RFP positive, GFP^+^RFP^+^) and red fluorescent puncta (GFP negative and RFP positive, GFP^−^RFP^+^). The data presented that compared with the Vector control, yellow puncta number significantly increased, while red puncta number significantly decreased after P301S‐tau overexpression (Figure 1b,c), indicating that P301S‐tau inhibited the fusion of autophagosomes and lysosomes. Furthermore, we found that the number of autophagosomes in P301S‐tau mice was markedly increased compared with WT mice by electron microscopy (Figure 1d,e). In short, P301S‐tau inhibited the generation of autophagosomes in the early stage of autophagy and hindered autophagosomes and lysosomes fusion in the late stage of autophagy, mainly in the impairments of autophagosomes and lysosomes fusion.

To explore the molecular mechanism of P301S‐tau induced autophagic impairment, we analyzed the mouse protein expression profile of the hippocampus of P301S‐tau mice. We found that the expression of autophagy or lysosome associated proteins was significantly abnormal compared with WT control, and TECPR1 protein level decreased (Figure S2a,b).

TECPR1 can act as a tether factor between autophagosomes and lysosomes to recruit autophagosomes, thereby promoting autophagosomes and lysosomes fusion and accelerating the process of autophagic degradation. Downregulation of TECPR1 protein levels were detected in the hippocampus of 9‐month‐old P301S‐tau mice by western blotting (Figure 1f,g) and immunohistochemistry assay (Figure 1h,i). Meanwhile, we injected AAV‐P301S‐tau‐GFP virus into the hippocampus of 2‐month‐old C57 mice, and also found a significant decreased TECPR1 protein level by western blotting (Figure 1j,k). By the way, we also detected the cell type distribution of TECPR1 in the normal hippocampus using neuronal markers (NeuN), microglial markers (Iba1), and astrocytes markers (GFAP) by immunofluorescence (Figure S3). The results showed that TECPR1 was detected in NeuN, Iba1, and GFAP positive cells, indicating that TECPR1 is widely expressed in the hippocampus.

### 2.2 TECPR1 activates autophagy by promoting the formation of autophagosomes

To explore the role of TECPR1 on P301S‐tau induced autophagic dysfunction, we transfected GFP‐TECPR1 or its vector plasmids into HEK293 cells. Western blotting data presented that TECPR1 significantly increased the level of LC3‐II protein, while the effect of TECPR1 on increasing LC3‐II protein level was markedly weakened after administering the autophagosome generation inhibitor 3‐Methyladenine (3‐MA) (Figure 1l,m). The immunofluorescence results confirmed that TECPR1 increased the number of LC3 puncta, which was offset by 3‐MA (Figure 1n,o). In addition, we overexpressed TECPR1 in HEK293 cells and subsequently treated with Baf A1 to inhibit autophagosomes and lysosomes fusion, thereby inhibiting autophagosome degradation, and we found that LC3‐II level in both groups increased after treatment with Baf A1, while the protein levels of LC3‐II were still higher in TECPR1 group than in Vector group (Figure 1p,q). The immunofluorescence results confirmed that TECPR1 increased LC3 puncta, and further increased LC3 puncta after Baf A1 treatment (Figure 1r,s). These results indicate that TECPR1 promotes autophagosome generation. In order to study the role of TECPR1 in late phase of autophagy, we transfected HA‐TECPR1 or its empty vector simultaneously with GFP‐RFP‐LC3 plasmids in HEK293 cells. The data presented that, overexpression of TECPR1 significantly increased the of red puncta (GFP^−^RFP^+^) number compared with the Vector control (Figure 1t,u). The above results suggest that TECPR1 can promote the fusion of autophagosomes and lysosomes. In summary, TECPR1 can activate autophagy in both early and late phase.

### 2.3 Overexpressing TECPR1 ameliorates cognitive deficits of P301S‐tau mice

**FIGURE 2:**
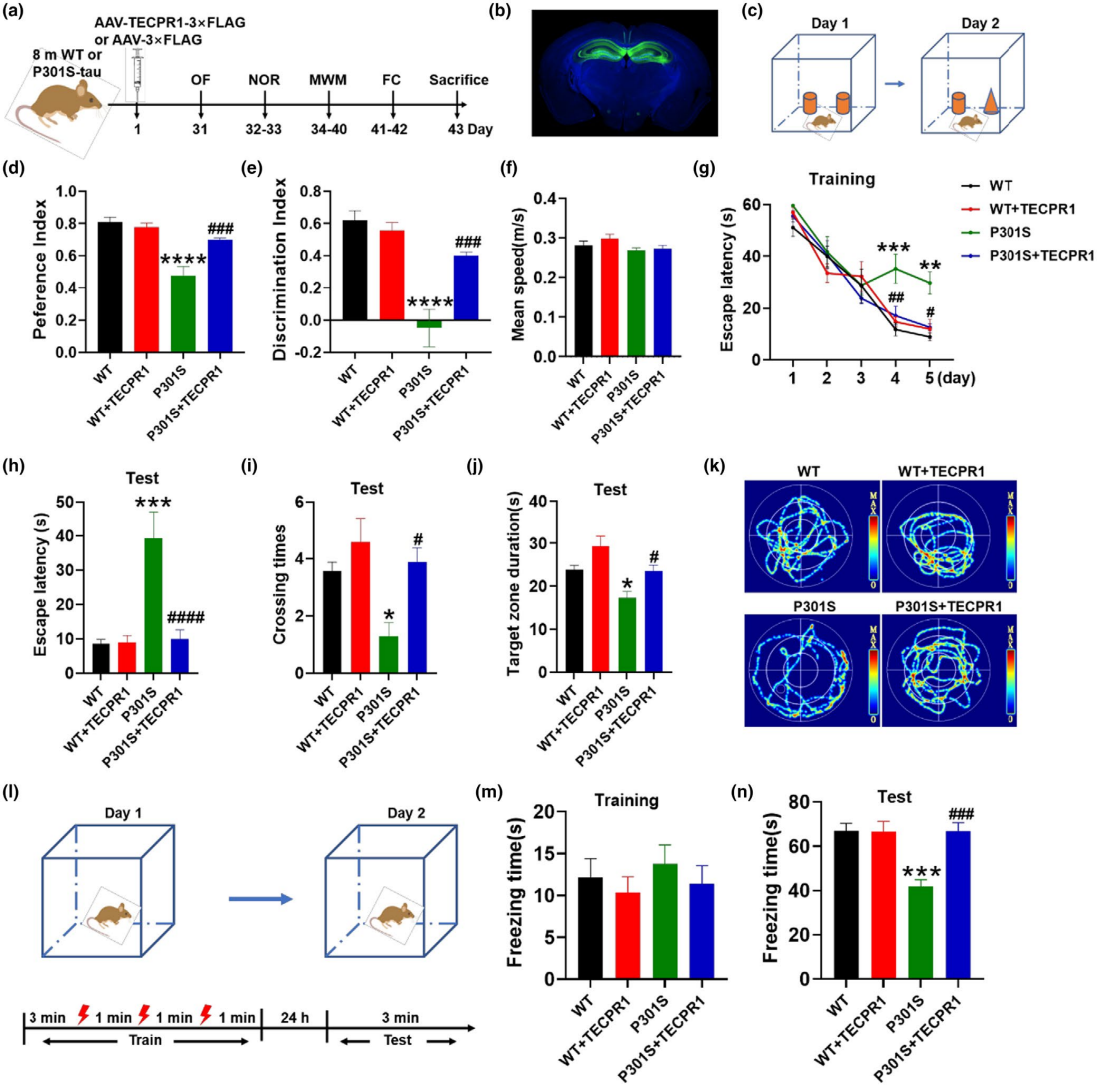
TECPR1 ameliorated cognitive deficits in P301S‐tau mice. AAV‐TECPR1‐3 × FLAG or AAV‐3 × FLAG virus was injected stereotaxically into the hippocampus of 8‐month‐old P301S‐tau mice or the age‐matched wild‐type littermates. One month later, the cognitive behavior was detected. (a) Diagram of the experimental procedure. (b) Virus infection areas were detected by immunofluorescence. (c–e) New object recognition test (NOR). (c) The schematic diagram for NOR. Preference (d) or discrimination index (e) during test in NOR. (f–k) Morris water maze test (MWM). (f) The swimming speed of mice. (g) The escape latency to platform during training phase. (h) The escape latency to platform during test phase, (i) Times across the platform, (j) Time in the third quadrant. (k) The representative swimming trace during test day. (l–n) Fear conditioning test (FC). (l) The schematic diagram for FC. (m) Freezing time in traing day. (n) Freezing time in test day. *N* = 7–8 mice each group. All data were presented as mean ± SEM. Two‐way repeated measures ANOVA test followed by Tukey's post hoc test for g, and One‐way ANOVA test followed by Tukey's post hoc test for others. *p < 0.05, **p < 0.01, ***p < 0.001, ****p < 0.0001 versus WT; #*p* < 0.05, ##*p* < 0.01, ###*p* < 0.001, ####*p* < 0.0001 versus P301S.

To investigate the effect of TECPR1 overexpression on cognitive function of P301S‐tau mice, we injected AAV‐TECPR1‐3 × FLAG or the vector control virus into the hippocampus of 8‐month‐old P301S‐tau transgenic mice (P301S) or the age‐matched wild‐type littermates (WT) through brain stereotactic injection. Behavioral testing was performed 1 month later (Figure 2a). The main infected areas of the virus were the hippocampal DG and CA3 regions (Figure 2b). First, we used open field test to detect whether the mice had anxiety and depression tendencies. The results showed that there were no significant differences in the total distance (Figure S4a), average speed (Figure S4b), and central area residence time (Figure S4c) between WT mice and P301S‐tau mice after overexpression of TECPR1, indicating that overexpression of TECPR1 did not alter the mental state of mice. The schematic of new object recognition was shown in Figure 2c. The data presented that compared with the WT control, the preference index for new objects and the discrimination index between new and old objects of P301S‐tau mice both significantly decreased. Overexpression of TECPR1 in P301S‐tau mice significantly increased the preference index and the discrimination index between new and old objects, while overexpression of TECPR1 did not change the preference and the discrimination index of WT mice (Figure 2d,e). In Morris water maze test, neither WT mice nor P301S‐tau mice showed significant changes in swimming speed after overexpression of TECPR 1 (Figure 2f). On the 4th and 5th training day, the latency of P301S‐tau mice to explore the platform was significantly longer than WT group, while the escape latency of P301S‐tau mice was significantly shorter after overexpression of TECPR1 (Figure 2g). Similarly, during the memory test phase, compared with the WT control, the latency of P301S mice was significantly prolonged, and the number of crossing the platform and the residence time in the quadrant where the platform was located were reduced significantly. However, these indicators were significantly reversed after overexpression of TECPR1 (Figure 2h–k). The schematic diagram of conditioned fear experiment was shown in Figure 2l. The results showed that overexpression of TECPR1 did not affect the freezing time of WT mice and P301S‐tau mice during the training phase (Figure 2m). During the test day, P301S‐tau mice showed significantly reduced freezing time compared with the WT control, and the freezing time of P301S‐tau mice increased significantly after overexpression of TECPR1 (Figure 2n). These data imply that overexpressing TECPR1 reverses cognitive impairments of P301S‐tau mice.

### 2.4 TECPR1 alleviates neuronal loss and improves synaptic plasticity of P301S‐tau mice

**FIGURE 3:**
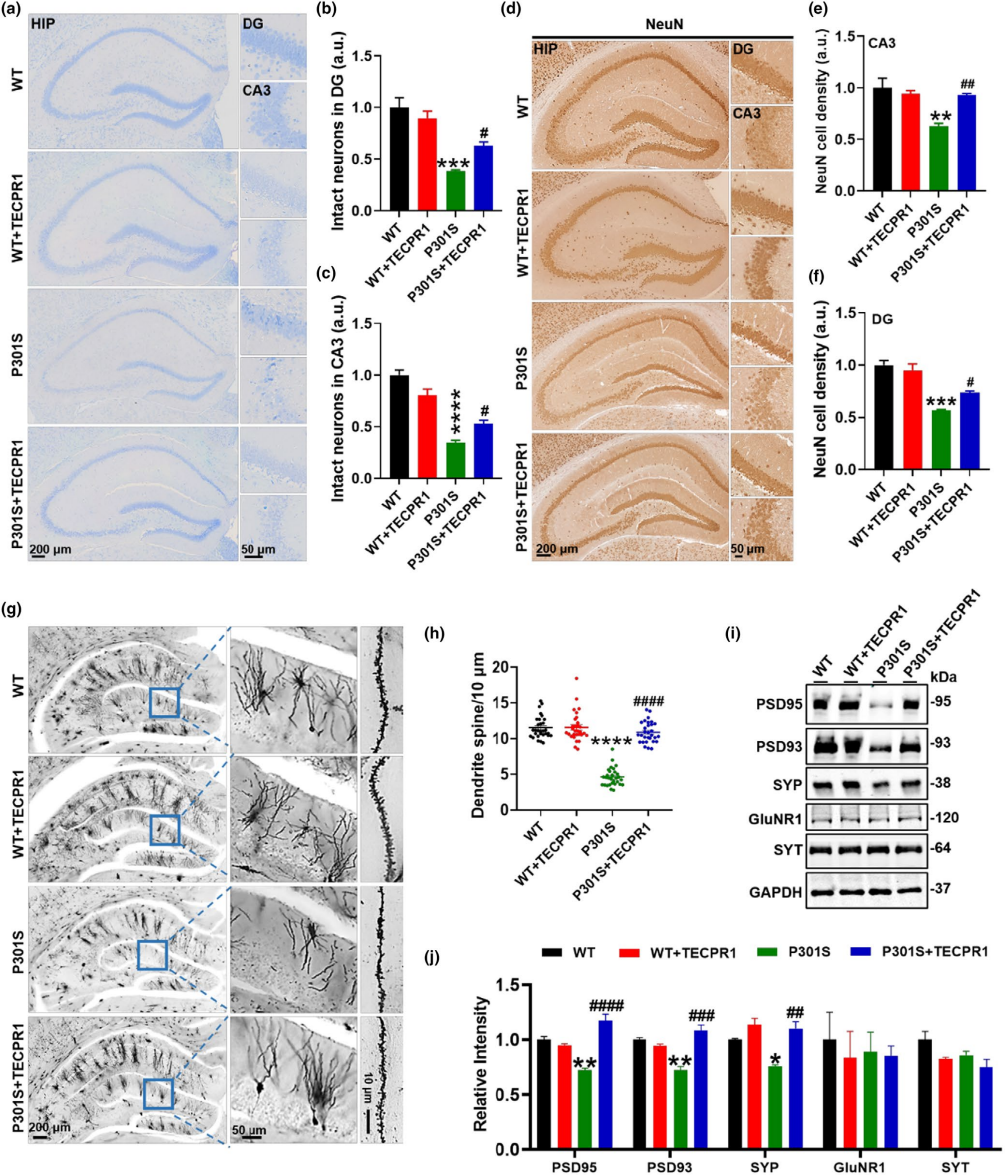
TECPR1 alleviated neuronal loss and improved synaptic plasticity of P301S‐tau mice. AAV‐TECPR1‐3 × FLAG or AAV‐3 × FLAG virus was injected stereotaxically into the hippocampus of 8‐month‐old P301S‐tau mice or the age‐matched wild‐type littermates for 1 month. (a–c) Representative images of Nissl staining (a), and quantitative analysis (b, c). (d–f) Representative images of immunohistochemistry staining with antibody anti‐NeuN (d), and quantitative analysis for the number of NeuN positive neurons (e, f). *N* = 3 each group. (g, h) Representative images of Golgi staining (g), quantitative analysis for the number of dendrite spine (h). *N* = 30 neurons from 3 mice for each group. (i, j) The synaptic related protein levels were detected by western blotting (i) and quantitative analysis (j). *N* = 3 each group. All data were presented as mean ± SEM, one‐way ANOVA test followed by Tukey's post hoc test. *p < 0.05, **p < 0.01, ***p < 0.001, ****p < 0.0001 versus WT; #*p* < 0.05, ##*p* < 0.01, ###*p* < 0.001, ####*p* < 0.0001 versus P301S.

To explore the underlying mechanisms of TECPR1 improved cognition, we first detected the number of neurons by Nissl staining, and found significant loss of hippocampal neurons in P301S‐tau mice. Overexpression of TECPR1 increased neuron number in the DG and CA3 regions of P301S‐tau mice (Figure 3a–c). Immunohistochemical detection of NeuN positive neurons determined that TECPR1 increased neuron number in the DG and CA3 regions of hippocampus in P301S‐tau mice (Figure 3d–f), while TECPR1 overexpressing in the hippocampus had no significant effect on the number of cortical neurons (Figure S5a). Golgi staining was used to detect the density of dendritic spines of neurons in the DG region of the hippocampus of mice. The results presented that, the number of neurons and the density of dendritic spines in the DG region of P301S group was significantly reduced compared with the WT group. The above data implied that, TECPR1 overexpression reversed the loss of neurons and the decrease dendritic spine density in the DG region of P301S‐tau mice (Figure 3g,h). Meanwhile, we detected the levels of synaptic related proteins and found that presynaptic protein SYP (synaptophysin) and postsynaptic proteins, including postsynaptic dense‐95 (PSD95) and postsynaptic dense‐93 (PSD93), significantly decreased in the P301S group. Overexpression of TECPR1 reversed the decrease in SYP, PSD95, and PSD93 proteins in the P301S group. The expression of synaptotagmin (SYT) and N‐methyl‐D‐aspartate receptor 1 (GluNR1) had no significant change (Figure 3i,j). These data suggested that overexpressing TECPR1 improves synaptic plasticity of P301S‐tau mice.

### 2.5 TECPR1 promotes tau protein degradation

**FIGURE 4:**
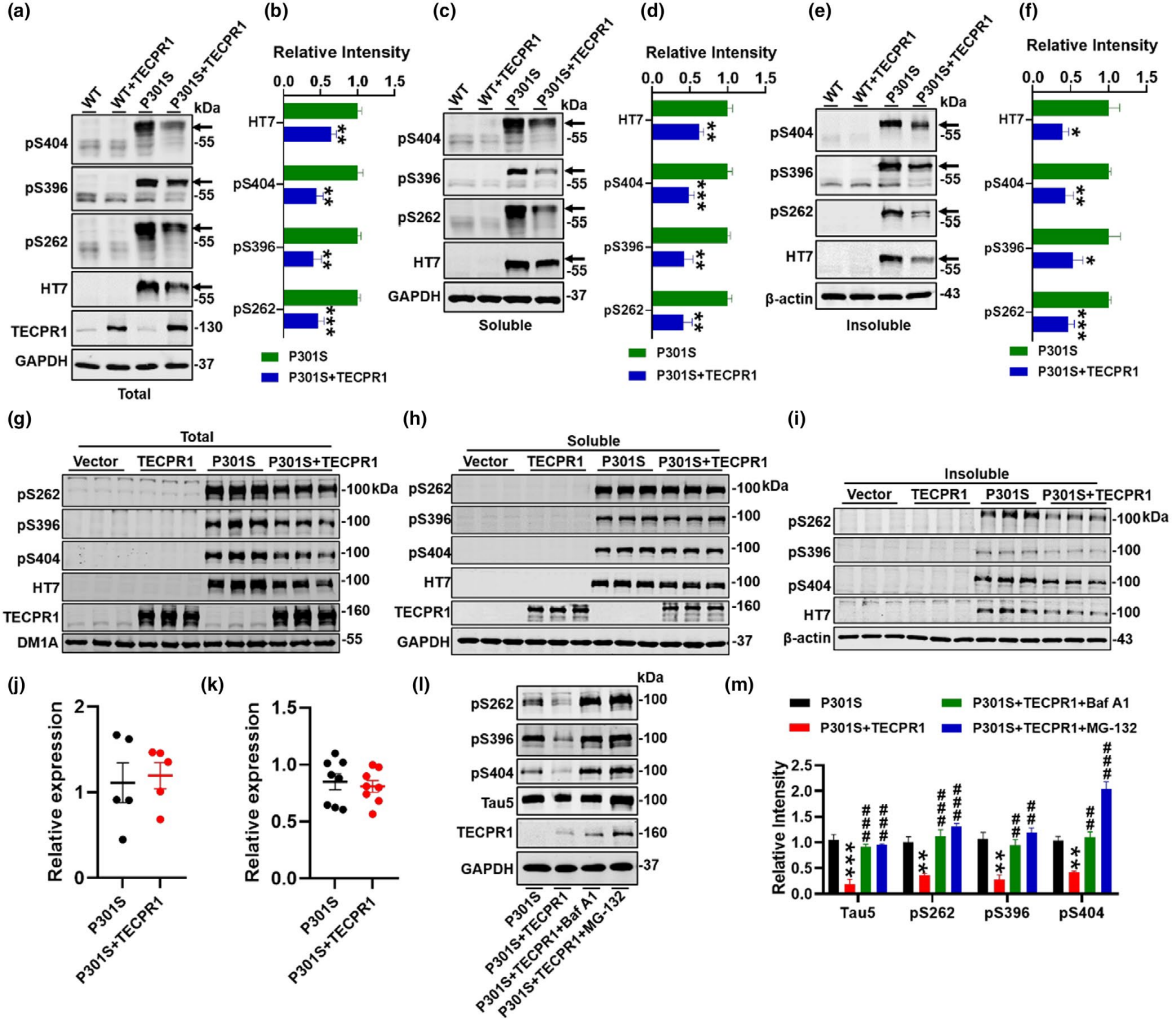
TECPR1 promoted tau degradation. AAV‐TECPR1‐3 × FLAG or AAV‐3 × FLAG virus was injected stereotaxically into the hippocampus of 8‐month‐old P301S‐tau mice or the age‐matched wild‐type littermates. (a–f) Total tau (HT7) and phosphorylated tau at Ser404, Ser396, Ser262 in total (a, b), soluble (c, d) and insoluble (e, f) fraction of hippocampus were detected by western blotting and quantitative analysis. The black arrow indicates P301S‐tau. *N* = 4–5 each group. (g–i) HEK293 cells were transfected with GFP‐P301S‐tau or/and CFP‐TECPR1 for 48 h, total tau (HT7) and phosphorylated tau at Ser404, Ser396, Ser262 in total (g), soluble (h) and insoluble (i) fraction were detected by western blotting. *N* = 3–6 each group. (j, k) Quantitative analysis of total tau (MAPT) mRNA levels of hippocampus in 9‐month‐old P301S‐tau mice with TECPR1 overexpression (AAV‐TECPR1‐3 × FLAG virus transfection) (j) and HEK293 cells overexpressing P301S‐tau and TECPR1 (k), *N* = 5–8 each group. (l, m) HEK293 cells were transfected with GFP‐P301S‐tau and CFP‐TECPR1 for 24 h, and then treated with Baf A1 (100 nM) or MG‐132 (5 μM) for 24 h, total tau (HT7) and phosphorylated tau at Ser404, Ser396, Ser262 were detected by western blotting (l) and quantitative analysis (m). *N* = 3 each group. All data were presented as mean ± SEM. One‐way ANOVA test followed by Tukey's post hoc test for (n), and unpaired *t*‐test for others. *p < 0.05, **p < 0.01, ***p < 0.001 versus P301S; ##*p* < 0.01, ###*p* < 0.001 versus P301S + TECPR1.

To further investigate the underlying mechanisms of TECPR1 ameliorated cognition, we detected the levels of total and phosphorylated tau proteins in the virus expressed site of hippocampus, and found that TECPR1 overexpression in P301S‐tau mice significantly decreased total and phosphorylated tau proteins (Figure 4a,b). Further studies on soluble or insoluble fractions have shown that TECPR1 overexpression reduced both soluble and insoluble tau (Figure 4c–f). As expected, the tau in the cortex had no change (Figure S5b,c). In HEK293 cells overexpressing P301S‐tau, TECPR1 overexpression also reduced total and phosphorylated tau in total soluble and insoluble fraction (Figure 4g–i, Figure S6a–c).

We validated that overexpression of TECPR1 can reduce total/phosphorylated tau levels both in vitro and in vivo, and then we explored its potential mechanisms. Similarly, we tested whether TECPR1 had an effect on influenced tau production transcription level. The mRNA levels of total tau (MAPT) were detected by qRT‐PCR, and we found that TECPR1 overexpression did not change MAPT mRNA level in cells expressing P301S‐tau or P301S‐tau mice (Figure 4j,k). Therefore, we co‐transfected P301S‐tau and/or TECPR1 in HEK293 cells and administered autophagy inhibitor Baf A1 and ubiquitin‐proteasome inhibitor MG‐132, respectively. And the results showed that after administering these two inhibitors, the effect of TECPR1 on reducing tau was reversed (Figure 4l,m).

### 2.6 TECPR1 rescues P301S‐tau‐induced autophagy deficits

**FIGURE 5:**
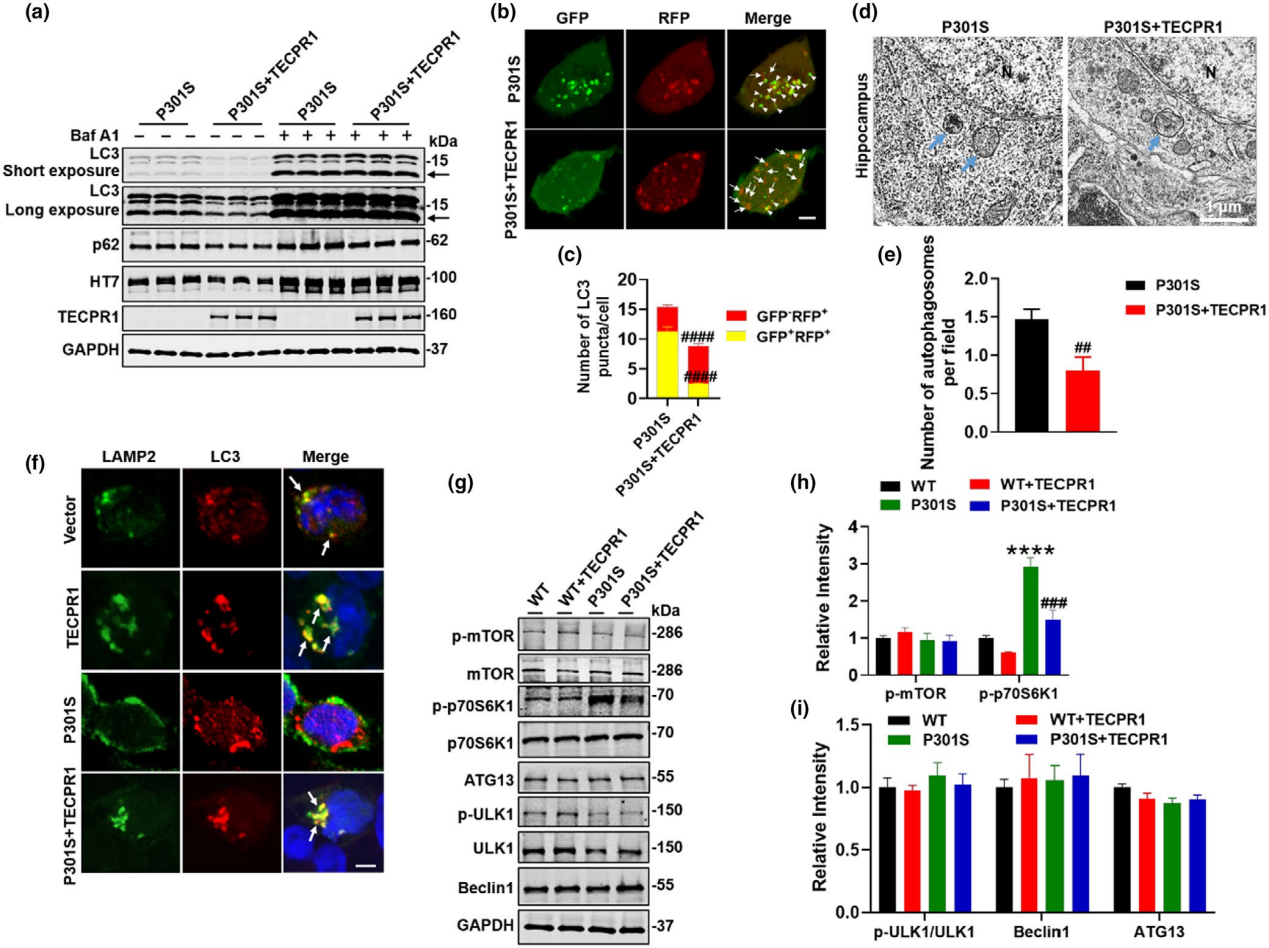
TECPR1 rescued P301S tau‐induced autophagy deficits. (a) HEK293 cells were transfected with GFP‐P301S‐tau and CFP‐TECPR1 for 24 h, and then treated with Baf A1 (100 nM) for 24 h, LC3‐II and p62 proteins were detected by western blotting. (b, c) HEK293 cells were transfected with 3 × FLAG‐P301S‐tau and HA‐TECPR1 and GFP‐RFP‐LC3 for 48 h, LC3 puncta of live cells were detected (b) and quantitative analysis (c). Arrow, GFP^−^RFP^+^ puncta; Arrowhead, GFP^+^RFP^+^ puncta. (d, e) Overexpression of TECPR1 markedly increased the number of autophagosomes compared with WT mice measured by transmission electron microscopy (d) and quantitative analysis (e). Blue arrow, autophagosome; *N*, nucleus; *N* = 3 mice/group. (f) HEK293 cells were transfected with GFP‐P301S‐tau or/and CFP‐TECPR1 for 48 h, co‐localization of LAMP2 and LC3 was measured by immunofluorescence. *N* = 3 independent experiments for each group. The arrow indicates co‐localization of LAMP2 and LC3. (g–i) AAV‐TECPR1‐3 × FLAG or AAV‐3 × FLAG virus was injected stereotaxically into the hippocampus of 8‐month‐old P301S‐tau mice or the age‐matched wild‐type littermates. The proteins of mTOR pathway were detected by western blotting (g) and quantitative analysis (h, i), *N* = 3 each group. All data were presented as mean ± SEM. One‐way ANOVA test followed by Tukey's post hoc test for e, g, and h, and Unpaired *t*‐test for c and e. ****p < 0.0001 versus WT; ##*p* < 0.01, ###*p* < 0.001, ####*p* < 0.0001  versus P301S.

As our data shown that, there is autophagic deficits in P301S‐tau mice, and TECPR1 activates autophagy, we focus on the autophagy pathway here. To explore the underlying mechanisms of TECPR1 overexpression reduced tau levels in P301S‐tau, we overexpressed TECPR1 in HEK293 cells overexpressing P301S‐tau. Western blotting showed that TECPR1 significantly reduced the LC3‐II and p62 levels (Figure 5a), and then, after Baf A1 was administered, we found an increase in LC3‐II level in the P301S group, while overexpressing TECPR1 promoted further elevation of LC3‐II level (Figure 5a), indicating that TECPR1 can promote autophagosome generation. Meanwhile, co‐transfection of HA‐TECPR1 and GFP‐RFP‐LC3 plasmid in cells overexpressing P301S‐tau revealed an obvious increase in the number of GFP^−^RFP^+^(red puncta) (Figure 5b,c). In addition, we found that overexpression of TECPR1 significantly decreased the number of autophagosomes in P301S‐tau mice (Figure 5d,e). Next, we transfected 3 × FLAG‐P301S‐tau and/or HA‐TECPR1 plasmids into HEK293 cells. It was found that overexpressing P301S‐tau significantly reduced the co‐localization of lysosomal marker (LAMP2) and autophagosome marker (LC3), while TECPR1 overexpression markedly increased the co‐localization of LAMP2 and LC3 (Figure 5f), and LAMP2 and HT7 (Figure S7). Furthermore, western blotting analysis showed that TECPR1 overexpression did not affect lysosome‐associated proteins LAMP1, LAPTM4a, ATP6V0D1, and ATP6V0D2 protein levels (Figure S8). In order to further explore the mechanism by which TECPR1 promoting autophagosome generation, we detected the mTORC1 (mechanistic target of rapamycin complex 1) pathway, which is the main negative regulator of early autophagy. We found that, the mTOR protein and its phosphorylated (Ser2448) levels did not show significant changes after overexpression of P301S‐tau only or with TECPR1, but the phosphorylated level of p70S6K1, the direct effector protein of mTORC1, was significantly upregulated in the hippocampus of P301S‐tau mice. Overexpression of TECPR1 reduced the phosphorylation level of p70S6K1 in P301S‐tau mice (Figure 5g,h). However, ATG13, ULK1, and ULK1 phosphorylation (Ser757) levels showed no significant changes (Figure 5g,i). These results suggest that TECPR1 promoted autophagosome generation by inhibiting the mTORC1 signaling pathway.

### 2.7 Inhibiting autophagosome generation by 3‐MA reverses the ameliorated effect of TECPR1 on cognitive function of P301S‐tau mice

**FIGURE 6:**
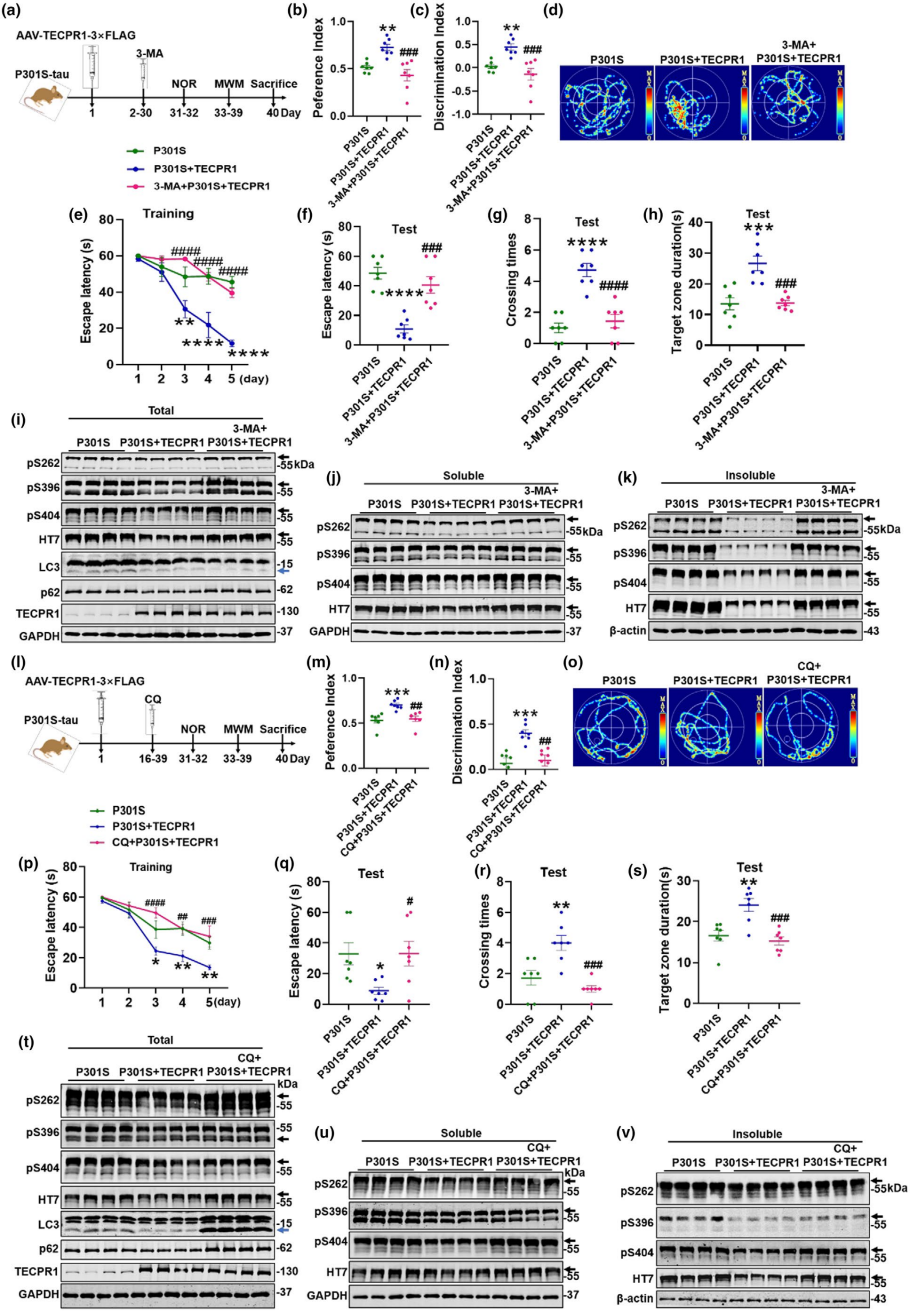
Inhibiting autophagy by 3‐MA or CQ offseted the ameliorated effect of TECPR1 on cognitive function of P301S‐tau mice. (a–k) AAV‐TECPR1‐3 × FLAG virus was injected stereotaxically into the hippocampus of 8‐month‐old P301S‐tau mice, followed by daily intraperitoneal injection of 3‐MA (45 mg/kg) for 30 days. (a) Diagram of the experimental procedure. (b, c) New object recognition test (NOR), preference (b) and discrimination index (c) during test in NOR. (d–h) Morris water maze test (MWM), (d) the escape latency to platform during training days, (e) the escape latency to platform during test day, (f) times across the platform, (g) time in the third quadrant. (h) The representative swimming trace during test day, *N* = 7 each group. (i–k) Total tau (HT7) and phosphorylated tau at Ser404, Ser396, Ser262 in total (i), soluble (j) and insoluble (k) fraction of hippocampus were detected by western blotting, *N* = 4 each group. (l–v) AAV‐TECPR1‐3 × FLAG virus was injected stereotaxically into the hippocampus of 8‐month‐old P301S‐tau mice, followed by daily intraperitoneal injection of CQ (50 mg/kg) for 15 days. (l) Diagram of the experimental procedure. (m, n) New object recognition test (NOR), preference (m) and discrimination index (n) during test in NOR. (o–s) Morris water maze test (MWM), (o) the escape latency to platform during training days, (p) the escape latency to platform during test day, (q) times across the platform, (r) time in the third quadrant. (s) The representative swimming trace during test day. *N* = 7 each group. (t–v) Total tau (HT7) and phosphorylated tau at Ser404, Ser396, Ser262 in total (t), soluble (u) and insoluble (v) fraction of hippocampus were detected by western blotting. *N* = 4 each group. The black arrow indicates P301S‐tau. The blue arrow indicates LC3‐II.All data were presented as mean ± SEM. Two‐way repeated measures ANOVA test followed by Tukey's post hoc test for e, p, and One‐way ANOVA test followed by Tukey's post hoc test for others. *p < 0.05, **p < 0.01, ***p < 0.001 ****, *p* < 0.0001 versus WT; #*p* < 0.05, ##*p* < 0.01, ###*p* < 0.001, ####*p* < 0.0001 versus P301S.

To verify that TECPR1 improves cognitive ability by promoting autophagosome generation, we injected AAV‐TECPR1‐3 × FLAG virus into the hippocampus of 8‐month‐old P301S‐tau mice, with administration 3‐MA for 30 days by intraperitoneal injection daily. After 1 month, we conducted cognitive related behavioral tests (Figure 6a). The results of new object recognition showed that 3‐MA significantly inhibited the improved effects of TECPR1 on the preference and discrimination index of P301S‐tau mice (Figure 6b,c). Morris water maze test showed that 3‐MA significantly reversed the effect of TECPR1 on shortening the latency of P301S‐tau mice to find platform during the training phase, and during the test phase, 3‐MA eliminated the ameliorated effects of TECPR1 in shortening escape latency, increasing crossing platform times, and prolonging duration in the quadrant where the platform is located (Figure 6d–h). Meanwhile, we detected the effect of 3‐MA on TECPR1‐induced decreased tau levels through western blotting. After TECPR1 overexpression in the hippocampus of P301S‐tau mice, both LC3‐II and p62 protein levels decreased, while 3‐MA further induced LC3‐II level decrease and p62 level increase (Figure 6i). 3‐MA reversed the effect of TECPR1 on reducing total tau, further measuring soluble and insoluble tau revealed 3‐MA mainly manifested as a stronger reversal effect on insoluble tau (Figure 6i–k, Figure S9a–c). In addition, 3‐MA also mainly reversed the degradation effect of TECPR1 on total and insoluble tau in HEK293 cells expressing P301S‐tau (Figure S9d–f).

### 2.8 Inhibiting the fusion of autophagosomes and lysosomes by CQ also counteracts the effect of TECPR1 on improving cognitive function of P301S‐tau mice

To further verify that TECPR1 improved cognitive ability by enhancing autophagosomes degradation, we injected AAV‐TECPR1‐3 × FLAG virus into the hippocampus of 8‐month‐old P301S‐tau mice, administered chloroquine (CQ) for 15 days by intraperitoneal injection to inhibit autophagosomes and lysosomes fusion at 15th day after virus injection and conducted cognitive related behavioral tests 1 month after virus infection (Figure 6l). The results of new object recognition test showed that CQ significantly inhibited the improvement of TECPR1 on the preference and discrimination index of P301S‐tau mice (Figure 6m,n). Morris water maze showed that CQ significantly reversed the ameliorated effect of TECPR1 on cognition of P301S‐tau mice (Figure 6o–s). Meanwhile, overexpression of TECPR1 in the hippocampus of P301S‐tau mice decreased both LC3‐II and p62 protein levels, while CQ induced LC3‐II and p62 levels increase (Figure 6t). CQ also reversed the effect of TECPR1 on reducing total, soluble and insoluble tau (Figure 6t–v, Figure S10a–c). In addition, we also got the similar data, that CQ reversed the effect of TECPR1 on reducing total tau, soluble and insoluble tau in vitro (Figure S10d–f).

## 3 DISCUSSION

Autophagy disorders have been shown in the brains of AD patients and animal models including htau, 3 × Tg, and APP/PS1 mice. The prior study showed that P301S‐tau expression disrupted lysosome pH by increasing Ca^2+^ release, therefore contributing to lysosome acidification defect (Tong et al., 2022). We also found in the previous research that overexpressing P301S‐tau in HEK293 cells suppressed autophagic activity by resulting in fusion deficits of autophagosomes and lysosomes (Feng et al., 2024). However, there are few studies on the relationship between P301S‐tau and autophagy in vivo. In this study, we demonstrated that P301S‐tau led to autophagy impairments by disturbing autophagosome formation by activation of mTOR signaling pathway. Moreover, the fusion between autophagosomes and lysosomes was suppressed by P301S‐tau, which proved via autophagy flux analysis. Hence, we firstly found that, P301S‐tau interfere with the early and late phase of the autophagy process including autophagosome generation, fusion between autophagosomes and lysosomes, which will enrich autophagy study in the field of tauopathies.

Compared with normal individuals, AD patients show a significant decrease in autophagy‐related gene expression, and led to tau and Aβ pathology (Tumurbaatar et al., 2023). In recent years, a lot of studies have also reported that activating autophagy to promote the clearance of Aβ plaque and tau aggregates can improve the learning and memory abilities of AD model mice (Heckmann et al., 2019; Song et al., 2020). These research findings demonstrate the potential of autophagy induction strategies in the treatment of AD. Therefore, we used proteomics to enrich autophagy and lysosome related proteins in P301S‐tau mice, and found that only the protein TECPR1 associated with both autophagy and lysosome was a significant decline in the P301S‐tau mice. Previous studies have shown that TECPR1 activates autophagy and is mainly involved in autophagy that selectively targets larger substrates, such as protein aggregates and damaged mitochondria (Chen et al., 2012; Ogawa et al., 2011; Ogawa & Sasakawa, 2011). The absence of TECPR1 blocked autophagosome maturation (Chen & Zhong, 2012). Moreover, TECPR1 localizes to lysosomal membranes and also acts as a tethering factor between autophagosomes and lysosomes, accelerating the autophagic degradation process by recruiting autophagosomes, thereby promoting the fusion of autophagosomes and lysosome. Here, we found that P301S‐tau overexpression obviously downregulated TECPR1 protein level in vitro and in vivo. However, overexpressing TECPR1 distinctly improved P301S‐tau induced autophagy disorder including autophagosome generation and the fusion of autophagosomes and lysosome.

mTOR is the main negative regulator of autophagy in the early stage (Galluzzi et al., 2014). Furthermore, we also detected the levels of mTOR and its common phosphorylation site Ser2448, and found that TECPR1 did not change the levels of mTOR and p‐mTOR (Ser2448). However, we detected that the phosphorylation level of mTORC1's direct effecter protein p70S6K1 significantly increased in P301S‐tau mice, while TECPR1 significantly decreased the level of p‐p70S6K1. Previous studies have shown that inhibiting the mTOR pathway to reduce the phosphorylation of p70S6K1 can also directly promote the formation of autophagosomes (Ittner & Ittner, 2018). This may be a potential mechanism for TECPR1 to promote the formation of autophagosomes.

When nutrition is sufficient, mTORC1 inhibits autophagy activity by inducing the phosphorylation of ULK1 at serine 757 (Jung et al., 2009). The inhibition of the mTORC1 pathway usually promotes the formation of the ULK1 complex (ULK1‐ATG13‐FIP200), which further promotes the formation of the VPS34 complex (VPS34‐ATG14L‐VPS15‐Beclin1) and thus promotes the initiation of autophagy (Backer, 2016; Galluzzi & Green, 2019). Therefore, we also detected the protein levels of downstream ULK1, ATG13, and Beclin1, but found no significant difference. These results suggest that TECPR1 may inhibit the mTORC1 pathway which presented by the phosphorylation level of p70S6K1 decrease to initiate autophagy. However, a more precise mechanism requires further in‐depth research.

One of the most popular views on how tau promotes the pathogenesis of tauopathy is that tau undergoes misfolding and oligomerization to form insoluble tau deposits, which gradually affecting neuronal basic functions and ultimately leading to neuronal death. Pathological tau disrupts synaptic composition and structure, leading to synaptic dysfunction and subsequent synaptic loss (Dejanovic et al., 2018; Ittner & Ittner, 2018). The most common symptom of AD is a decline in learning and memory abilities. The synaptic connections between neurons are dynamic and plastic, which is the foundation of learning and memory. In this study, overexpression of TECPR1 obviously improved learning and memory in P301S‐tau mice, reduced tau levels in total, soluble, and insoluble fragments both in vitro and in vivo, and significantly reversed P301S‐tau induced neuronal loss, and decreased dendritic spine density and synaptic associated proteins. These results suggest that TECPR1 overexpression ameliorates tau pathology and synaptic damage in P301S‐tau mice, which may be the basis for TECPR1's improvement learning and memory impairment induced by P301S‐tau.

As showed in the Figure 4l,m, TECPR1 overexpression degraded tau not only by autophagy but also by proteasome. We also found that the phosphorylation levels of p70S6K1 was markedly inhibited by TECPR1 overexpression in P301S‐tau mice, which suggested mTOR activity inhibition. Previous research indicated that mTOR inhibition would activate protein degradation by upregulating both ubiquitin‐proteasome system and autophagy in cultured cells (Zhao et al., 2015). Here, we speculate that TECPR1 may also activate the ubiquitin‐proteasome system to degrade tau protein. Moreover, we are currently conducting relevant research on the role of TECPR1 in the proteasome pathway.

To further determine that TECPR1 improves P301S‐tau induced learning and memory impairment by activating autophagy pathway, we overexpressed TECPR1 in the P301S‐tau hippocampus combined with autophagy initiation inhibitor 3‐MA and late stage autophagy inhibitor CQ treatment, respectively. The results showed that both 3‐MA and CQ treatments reversed the effect of TECPR1 on improving tau pathology and learning and memory in P301S‐tau mice. These data confirm that TECPR1 overexpression accelerates tau protein degradation and improves cognitive function in P301S‐tau mice by activating autophagy.

By the way, the increase in yellow LC3 puncta in the P301S group could potentially be attributed to an elevation in lysosomal pH, as previous studies have demonstrated lysosomal acidification defects in P301S‐tau transgenic mice (Tong et al., 2022). However, we observed a significant reduction in LC3 and LAMP2 colocalization in the P301S group (Figure 5f), suggesting a decrease in autophagosome‐lysosome fusion. Moreover, proteomics data revealed that some lysosome associated protein levels changed, our western blotting analysis of proteins related to lysosomal fusion/acidification showed no significant differences among the four groups (Figure S8). Therefore, though we could not exclude the possibility that an increase in lysosomal pH, our results suggest that the accumulation of yellow LC3 puncta in the context of P301S‐tau overexpression is more likely due to the impaired autophagosome‐lysosome fusion.

In summary, we found that P301S‐tau downregulated TECPR1 protein levels, leading to autophagy dysfunction by activating the mTOR signaling pathway, and exacerbating tau accumulation, ultimately resulting in synaptic plasticity impairments and cognitive deficits. Overexpressing TECPR1 inhibited the activity of the mTOR pathway to facilitate the generation of autophagosomes, and promote the fusion of autophagosomes and lysosome, thereby accelerating tau degradation, improving synaptic plasticity and cognition in P301S‐tau transgenic mice. TECPR1 may be a new target for treating AD.

## 4 EXPERIMENTAL PROCEDURES

### 4.1 Antibodies, plasmids, and viruses

The used antibodies were listed in the Table S1. pEGFP‐C1‐P301S‐tau and empty plasmid pEGFP‐C1‐Vector were constructed and synthesized by OBiO Technology (Shanghai) Corp., Ltd., 3 × FLAG‐P301S‐tau and empty plasmid were synthesized by Tsingke Biotechnology Co., Ltd., pCMV‐SPORT6‐TECPR1 (human) was purchased from Miaoling Biology, and then after modification of the vector and label by OBiO Technology (Shanghai) Corp., Ltd., the following plasmid was synthesized: CFP‐TECPR1, GFP‐TECPR1, HA‐TECPR1. GFP‐RFP‐LC3 plasmid was gifted by Professor Qing Tian in our research department; mCherry‐LC3 was gifted by Professor Xiangnan Zhang from Zhejiang University. pAAV‐hSyn‐P301S‐tau‐EGFP‐3 × FLAG and pAAV‐hSyn‐EGFP‐3 × FLAG viruses were synthesized by OBiO Technology (Shanghai) Corp., Ltd., pAAV‐hSyn‐TECPR1‐3 × FLAG and pAAV‐hSyn‐3 × FLAG viruses were synthesized by Shanghai Genechem Co., Ltd.

### 4.2 Drug administration

For animals: On the second day of virus injection, 3‐Methyladenine (3‐MA, Med Chem Express, HY‐19312) was administered intraperitoneally with a dose of 45 mg/kg for 1 month. After 15 days of virus injection, Chloroquine (CQ, Sigma, C6628) was administered intraperitoneally with a dose of 50 mg/kg for 15 days.

For cells: After 24 h of transfection with plasmids, Baf A1 (100 nM), 3‐MA (5 mM), MG‐132 (5 μM) or CQ (20 μM) was administered to cells for 24 h. The cells were cultured in a humidified atmosphere of 5% CO_2_ in air at 37°C.

### 4.3 Novel object recognition (NOR)

On the first day, the animals were placed in a white uncovered plastic box (40 × 40 × 40 cm), with two objects placed at the bottom of the box. The animals were allowed to explore for 5 min freely, and their exploration time for each object was recorded. On the second day, one of the objects was substituted with a new one, and the percentage of mice exploring new objects was calculated. At the end of each mouse experiment, the box needs to be wiped with 75% ethanol to prevent odor interference with subsequent animal behavior.

### 4.4 Morris water maze (MWM)

The experimental process is divided into training days (1–5 days) and test day (7th day). The experiment used a circular water pool (with a diameter of 120 cm and a height of 70 cm), which divided into four quadrants. A cylindrical platform (with a diameter of 10 cm and a height of 58 cm) was placed in the center of the third quadrant of the pool, and the water surface was kept 1.2 cm above the platform. The water temperature was maintained at around 23°C. The animal was placed in the opaque water at the edge of the pool wall in the middle of quadrants 1, 2, and 4, respectively. If the mouse reaches the platform within 60 s, it is recorded as the escape latency. If the animal did not find the platform within 60 s, the escape latency was recorded as 60 s and the experimenter guided the animal to reach and stay on the platform for 20 s. On the 7th day, after removed the platform, the experimenter detected the animal's memory abilities. The indicators during the test phase including escape latency, crossing platform times, duration in third quadrant were recorded.

### 4.5 Fear conditioning

On the first day, the animals were placed in an electric shock box, with the first 3 min as the adaptation period. After the adaptation period, a foot shock (intensity: 1 mA; duration: 2 s) was administered, a total of three shocks, with a time interval of 1 min between each two shocks. On the second day, the animals were placed in an electric shock box for 3 min without electrical stimulation. If mice have memory of electric shock scenes, they develop a fear response, leading to stiff behavior (Freezing), and the software automatically records the time of each freezing. At the end of each mouse experiment, the box needs to be wiped with 75% ethanol to prevent odor interference with subsequent mouse behavior.

### 4.6 Protein extraction

For cells: the cells were collected after washed with PBS twice to remove the cell culture medium. After completely discarding the PBS, the cells were added cell lysis buffer (Beyotime, P0013) containing PMSF (Sigma, P7626) and Phosphatase Inhibitor Cocktail (MCE, HY‐K0021) and 4 × SDS‐PAGE buffer. After the sample was fully cracked on ice, the protein was collected and boiled for 10 min, and then sonicated 20 times. Next, the sample was centrifuged at 12000 *g* for 5 min at 4°C, and the supernatant was collected.

For animals: After the mouse was euthanized, hippocampal tissue was taken and added with a homogenate containing PMSF and phosphatase inhibitors and 4 × SDS‐PAGE buffer. After thorough homogenization, it was boiled for 10 min and sonicated. Then, the sample was centrifuged at 12000 *g* for 5 min at 4°C. The supernatant was collected, which is the total cell protein.

### 4.7 Extraction of soluble and insoluble tau

After thoroughly homogenized the hippocampus with RIPA buffer (Beyotime, P0013B) containing PMSF and phosphatase inhibitors, or the cell samples were thoroughly lysed using the same lysis solution, the homogenate or cell lysis centrifuged at 13000 *g* for 20 min at 4°C, and then, the supernatant were collected and designated as the soluble fraction. The remaining precipitate was homogenized twice and centrifuged at 13000 *g* for 20 min at 4°C. After suspended and incubated with 70% formic acid (1:1 (*w*: *v*)) at 4°C with stirring overnight, the sample was centrifuged at 18000 *g* for 20 min at 4°C, and then the supernatant was collected. After evaporated formic acid, the precipitate was dissolved in 1 × SDS‐PAGE sample buffer, which was designated as the insoluble fraction.

### 4.8 Nissl's staining, immunofluorescence (IF) and immunohistochemistry (IHC)

Paraffin embedded brain slices are first dewaxed with xylene and subjected to gradient alcohol (100%–100%–95%–90%–80%–75%) hydration. Nissl staining was performed according to the instructions of the reagent kit (Servicebio, G1036). Specifically, after dewaxed and hydrated, brain slices were incubated with Nissl staining solution for 5–10 min, and then subsequently rinsed with tap water. After the brain slices were dried at room temperature, slide was sealed with neutral resin. Finally, images were obtained by brightfield microscope (Pannoramic SCAN, 3D Histech, Hungary).

Brain slices were boiled in citrate buffer for 20 min for antigen repair. After the brain slices were washed with PBS buffer containing 0.1% Triton X100 (PBST) three times, they were incubated with membrane breaking blocking solution (0.5% Triton X100 PBS + 5% BSA) for 40 min. Thereafter, these brain slices were incubated with the primary antibody overnight at 4°C, subsequently washed with PBST three times and then incubated with the secondary antibody for 1 h. Finally, these brain slices were incubated with 4′,6‐diamidino‐2‐phenylindole (DAPI) for 10 min at room temperature and acquired images by confocal fluorescent microscope (ANDOR, UK).

After performed dewaxing and hydration, brain slices were boiled in citrate buffer for 20 min for antigen repair, and then washed three times with PBST, the immunohistochemistry was performed by SV hypersensitive two‐step histochemical Kit (Boster, SV0001, SV0002, SV0003). Firstly, the brain slices were incubated with 3% H_2_O_2_ at room temperature for 20 min, and then washed three times by PBST. Afterwards, the brain slices were incubated with membrane breaking blocking solution for 1 h, and subsequently incubated with primary antibody overnight at 4°C. The slices were washed and then incubated with polymerized HRP labeled IgG. DAB staining was performed, and gradient alcohol (75%–90%–95%–100%–100%) dehydration and xylene transparency were carried out. Finally, these slices were sealed with neutral resin. We obtained the images by brightfield microscope (Pannoramic SCAN, 3D Histech, Hungary), which were analyzed by Image J Software.

### 4.9 Quantitative real‐time PCR

The total RNA was extracted using FreeZol Reagent (Vazyme, R711‐01, China). Reverse transcription was accomplished by HiScript IV RT SuperMix for qPCR kit (Vazyme, R423‐01, China). The real‐time quantitative PCR were performed with SYBR green kit (Vazyme, Q712‐02, China). The mRNA level of the objective gene was normalized by beta‐actin. All sequences of primers including MAPT and β‐actin referred to the literature we published in the past (Wang et al., 2021, 2024). Primers used for qRT PCR were as following: MAPT forward primer 5'‐CAGCTCCGGCACCAACAG‐3′ and reverse primer 5'‐CCTGGTTCAAAGTTCACCTGAT‐3′; β‐actin forward primer 5'‐GAGACCTTCAACACCCCAGC‐3′ and reverse primer 5'‐GGAGAGCATAGCCCTCGTAGAT‐3′;

### 4.10 Statistical analyses

All data were presented as the mean ± SEM and analyzed by the GraphPad Prism 8.0 (GraphPad Software, United States). The two‐way repeated measures analysis of variance (ANOVA) tests was used to analyze the training period of Morris water maze. One‐way ANOVA followed by Tukey's multiple comparison tests was used for statistical analysis of four groups. Statistical analyses of two‐group comparisons were performed by unpaired t test. The statistically significant difference was set at *p* < 0.05.

## AUTHOR CONTRIBUTIONS

T.L. and R.J.L. performed most of the experiments. Bioinformation analysis was completed X.L. and X.F.Y. The statistics analysis of this study was accomplished by Y.H. B.G.Z. and X.L. Animal experiments of mice were accomplished by X.X.R. and J.Z. The manuscript was written by T.L. and revised by G.P.L. These experiments designed by G.P.L., G.L. and J.Z.W.

## CONFLICT OF INTEREST STATEMENT

The authors declare that they have no competing interests.

## Supporting information


**Data S1:** Supporting Information.

## Data Availability

The data supporting the results of current study are available from the corresponding author.
