# Supplementary material for: Overexpression of TECPR1 improved cognitive function of P301S‐tau mice via activation of autophagy in the early and late process
Source: Aging Cell. 2024 Nov 7;24(3):e14404. doi: 10.1111/acel.14404 (PMC11896361; doi:10.1111/acel.14404)
Supplement: Supplementary file 1 — Data S1: Supporting Information. [file ACEL-24-e14404-s001.docx]

**Supplementary Materials for**

**Overexpression of TECPR1 improved cognitive function of P301S-tau mice via activation of autophagy in the early and late process**

Ting Li *et al.*

*Corresponding author: Gongping Liu, Email: liugp111@mail.hust.edu.cn

**Materials and Methods**

*1.1 Cell culture and transfection*

Human embryonic kidney 293 (HEK293) cells were cultured in Dulbecco’s modified Eagle’s medium (DMEM, Gibco, C11995500BT) containing 10% fetal bovine serum (FBS, VivaCell, C04001-050). The cells were plated onto 6-well or 12-well plates or glass bottom cell culture dish evenly, and plasmids were transfected using Lipofectamine^TM^ 3000 (Lipo3000, Thermo Fisher, L3000150) following the manufacturer’s instructions. All cultures were maintained in a humidified atmosphere of 5% CO_2_ in air at 37 °C.

*1.2 Animals and stereotactic injection*

P301S transgenic mice [B6; C3-Tg (Prnp-MAPT/P301S) PS19Vle/J], wild-type littermates and wild-type C57BL/6 mice (2-month-old, male) were purchased from Beijing Shulaibao Company in accordance with the law of the People’s Republic of China on Animal and Plant Inspection and Quarantine. The gender of mice used in this study was male. All mice were kept at a specific pathogen-free environment feeding center, with a 12-h light/dark cycle, an appropriate temperature and free food and water intake.

Mice were anesthetized with isoflurane and injected with the virus using the German KOPF brain stereotaxic device. The injection coordinates are based on the anterior fontanelle as the origin: DG (posterior: 1.9 mm, lateral: ± 1.1 mm, ventral -2.0). Behavioral tests were conducted one month after injection of the virus.

*1.3 Open field (OF)*

The mice were placed in a white uncovered plastic box measuring 40 cm×40 cm×40 cm and explored for 5 min. The total distance of movement, average movement speed, and time spent in the central area were recorded to evaluate the mental state and motor ability of the mice. If the mouse has a tendency towards anxiety or depression, there may be an increase in the total distance of movement and a decrease in the distance of movement at the center. At the end of each mouse experiment, the box needs to be wiped with 75% ethanol to prevent odor interference with subsequent mouse behavior.

*1.4 Western blotting*

The protein concentration of all samples was measured using the BCA kit (Thermo Fisher, 23225). According to the protein concentration of the sample, they were packed in the gel in equal quantities. After protein samples were separated by SDS-polyacrylamide gel electrophoresis for 1-2 h, the gel was transferred to the nitrocellulose (NC) membrane (Cytiva, 10600002). The NC membrane was incubated with 5% skim milk at room temperature for 1 h to block nonspecific binding, and subsequently incubated the primary antibody at 4 °C overnight. After the NC membrane was washed with TBS buffer containing Tween 20 three times, which was incubated the secondary antibody for 1 h, and finally washed with TBST three times. We used the Odyssey Infrared Imaging System (LI-COR Biosciences) to visualize the immunoreactive bands, which were analyzed by Image J Software.

*1.5 Golgi staining*

The Rapid Golgi Stain Kit (FD neurotechnologies, PK401) was used according the manufacturer’s instructions. Briefly, the intact brains were immersed in a special fluid (FD Solutions A : B = 1:1, vol/vol, mixed for 24 h in advance) for 4 weeks. Then, the brains were transferred to FD Solution C and incubated at 4 °C for one week. Subsequently, the brains were sliced into 100 μm brain slices by a vibrate microtome. These brain slices were attached to slides and rinsed by distilled water (2 min each, twice). Afterward, these slices were transferred to a prepared solution (FD Solutions D : E:distilled water = 1:1:2, vol/vol/vol) for 10 min. Subsequently, sections were dehydrated in gradient alcohol (50%, 75%, 95% and 100% ethanol) for 5 min each. Finally, they were permeabilized in xylene. The images of neurons in the hippocampal DG region were acquired with an Olympus BX60.

*1.6 Mass spectrometry analysis*

The hippocampal tissues were lysed in 8 M urea buffer and ultrasonicated in ice water. After the lysates were kept on ice for 20 min, they were centrifuged at 12,000 g, 4 °C for 15 min. The supernatant was by collected. The protein concentration was detected by the NanoDrop 3300 (Thermo Fisher Scientific, United States). After each sample with 50 µg protein was digested, they were labeled with TMT (Thermo Fisher, 90111), and then isolated by High Performance Liquid Chromatography (UltiMate 3000; Thermo Fisher Scientific, United States). Each sample was separated into 15 fractions, which were dried and dissolved in 20 ml 0.1% formic acid. The prepared polypeptide was further analyzed by liquid chromatography (LC)-mass spectrometry (MS)/MS. The quadrupole orbitrap mass spectrometer (Q-Exactive, Thermo Scientific, United States) was used to collect and analyze ionized peptides. All MS/MS spectra were searched with the uniprot mouse database by Proteome Discoverer 2.6 software. The LC-MS/MS data were deposited to the ProteomeXchange Consortium with the dataset identifier PXD029068. The criteria that are defined as differentially expressed proteins (DEPs) are *p* < 0.05 and fold change > 1.2 or < 0.83.

*1.7 Transmission electron microscopy*

The brains were removed and fixated in a 2.5 % glutaraldehyde solution for 24 h. Then, the brains were cut into 60 mm thick slices by a vibratome and brain slices including the hippocampal region were collected. Subsequently, the slices were transferred in 1% cesium tetroxide solution to postfixation for 1 h, and then, dehydrated in graded ethanol (30%, 50%, 70%, 80%, 95%, 100%, 100% ethanol), and embedded in epoxy resin. The above embedded samples were polymerized at 60 ℃ for 48 h. The samples were sliced 70 nm by ultra-thin microtome, and stained with 2% uranyl acetate for 8 min. After rinsing with 70% ethanol three times, they were washed by distilled water three times and then stained with 2.6% lead citrate solution. Finally, the Hitachi 7100 electron microscope (Nikon, Tokyo, Japan) was used to examine the number of autophagosome in the hippocampus.

**Supplementary Figures legends**


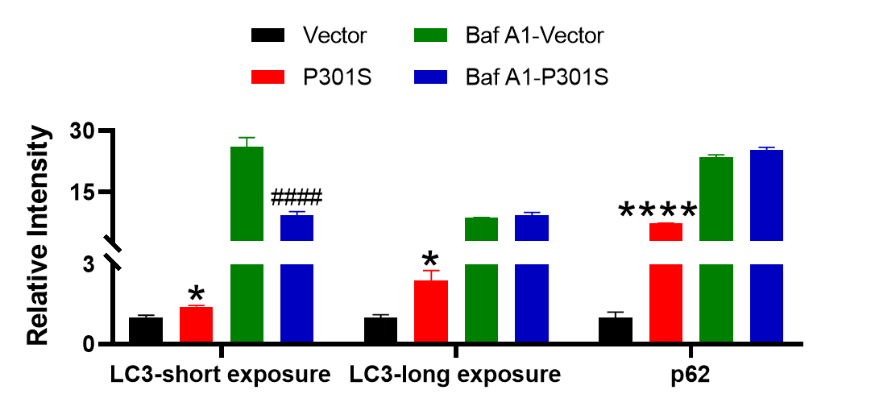


**Figure S1. P301S-tau induced autophagy defects.** Quantitative analysis for Figure 1A. N=3 each group. All data were presented as mean ± SEM. *, *p* < 0.05, ****, *p* < 0.0001, vs. Vector; ####, *p* < 0.0001 vs. P301S.


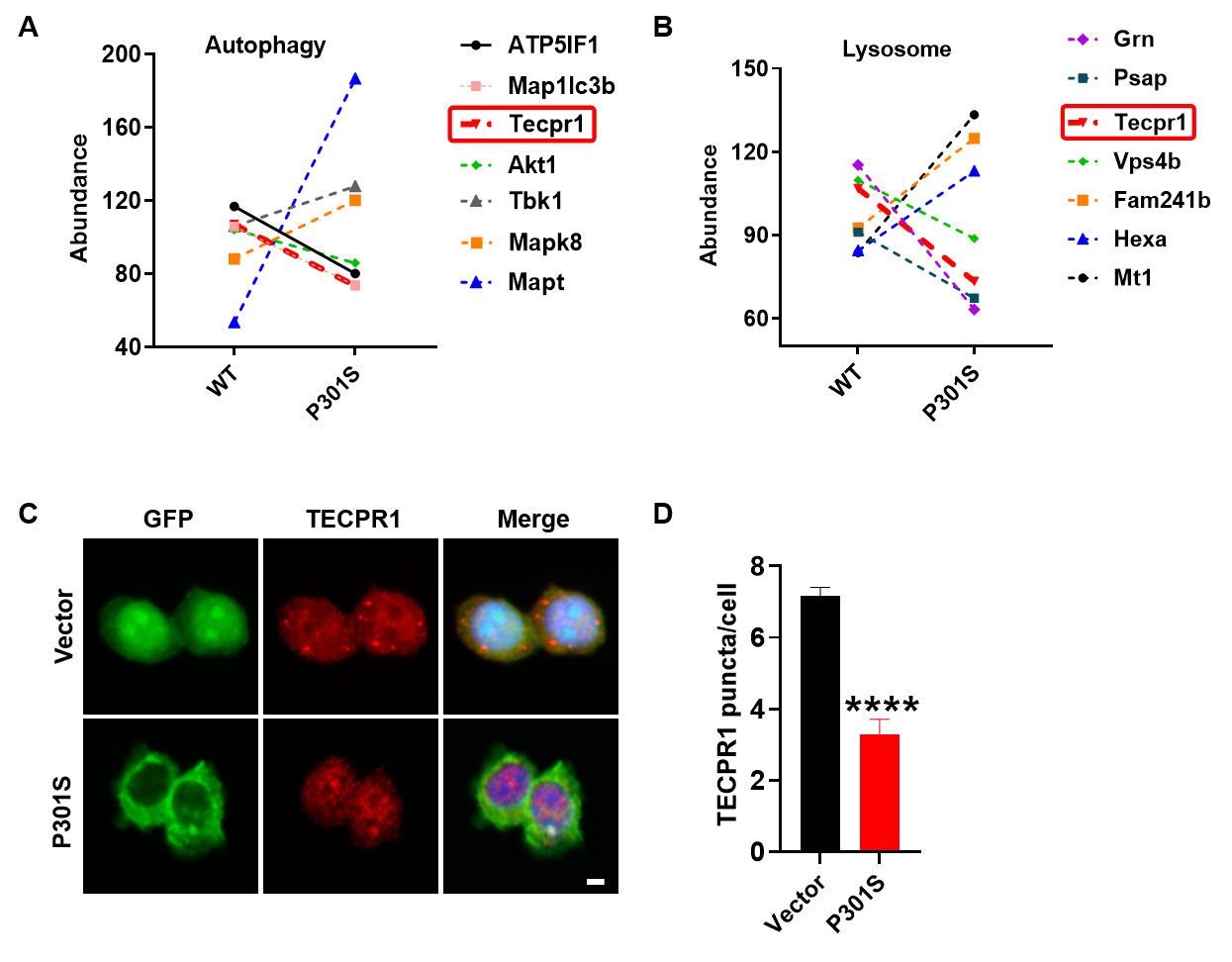


**Figure S2.** **GO analysis to classify autophagy and lysosome related proteins in the hippocampus of P301S-tau mice.** **(A)** autophagy, **(B)** lysosome. The change of each protein is shown as an average and is connected by dotted lines.


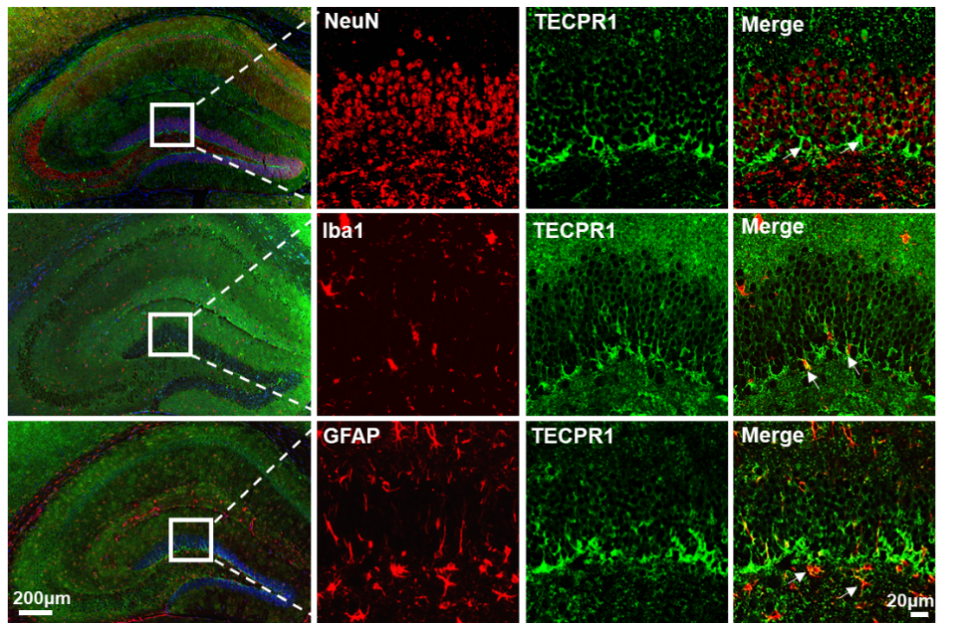


**Figure S3. TECPR1 is widely distributed in the hippocampus.** The expression of TECPR1 in NeuN, Iba1 and GFAP positive cells in the hippocampus of 2-month-old C57 mice was detected by immunofluorescence. The white arrow showed the expression of TECPR1 in the corresponding positive cells.


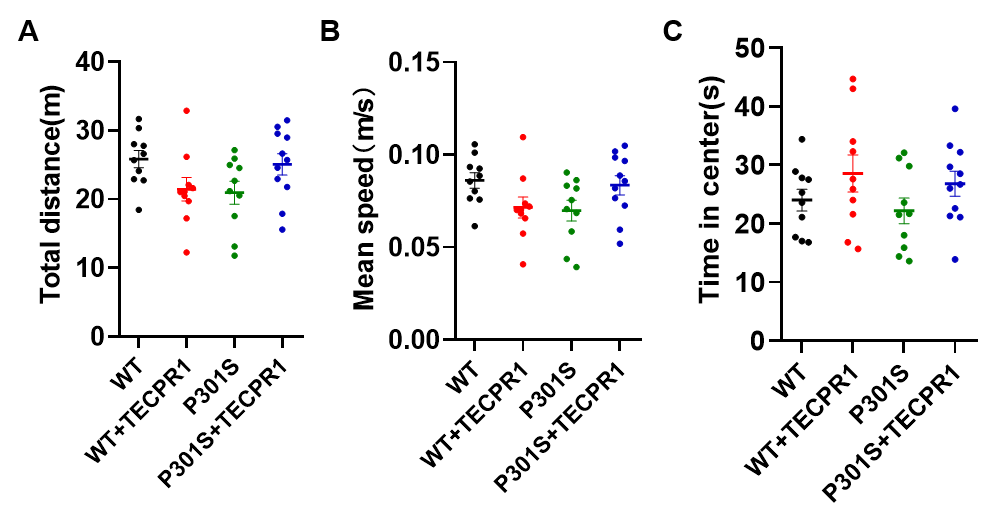


**Figure S4.** **TECPR1 did not alter the mental state of mice.** AAV-hSyn-TECPR1-3×FLAG or AAV-hsyn-3×FLAG virus was injected stereotaxically into the hippocampus of P301S-tau mice or the age-matched wild-type littermates, open field test (OF) was used to detected the behavior. **(A)** The total distance of mice in the open field test, **(B)** average movement speed of mice, **(C)** the duration of mice stayed in the center. N=9-10 each group. All data were presented as mean ± SEM. One-way ANOVA test followed by Tukey’s post hoc test.


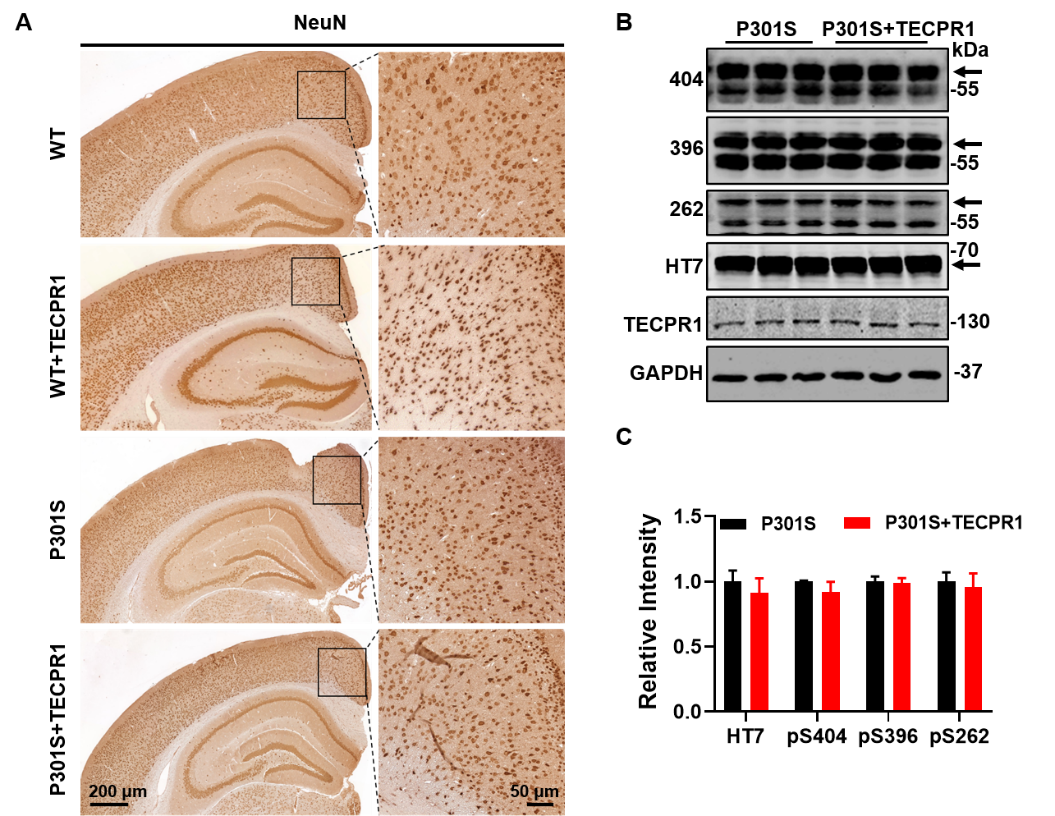


**Figure S5. The number of neurons or the levels of tau protein in the cortex had no sinificant change while TECPR1 overexpression in the hippocampus.** AAV-TECPR1-3×FLAG or AAV-3×FLAG virus was injected stereotaxically into the hippocampus of 8-month-old P301S-tau mice or the age-matched wild-type littermates for one month. **(A)** Representative images of cortical neurons stained using immunohistochemistry with the anti-NeuN antibody. **(B, C)** Total tau (HT7) and phosphorylated tau at Ser404, Ser396, or Ser262 site of the cortex were detected by western blotting **(B)** and quantitative analysis **(C)**. The black arrow indicates P301S-tau. N=3 each group. All data were presented as mean ± SEM. Unpaired t-test for all data.


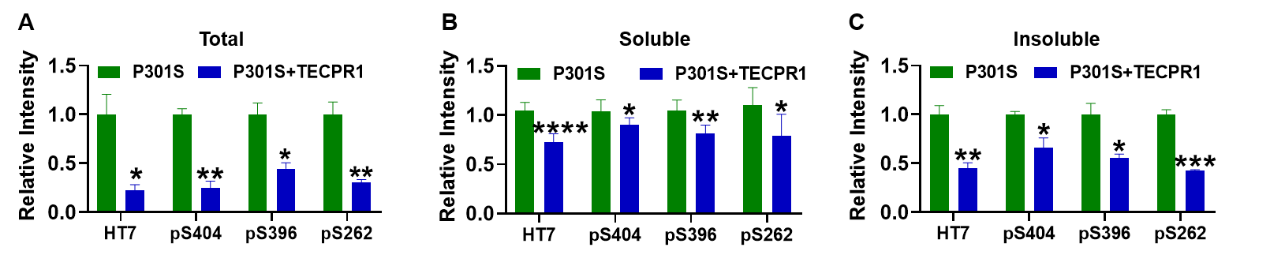


**Figure S6. TECPR1 promoted tau degradation.** Quantitative analysis of total tau (HT7) and phosphorylated tau at Ser404, Ser396, Ser202 in total **(A)**, soluble **(B)** and insoluble fraction **(C)** while TECPR1 overexpression in HEK293 cells overexpressing P301S-tau, western blotting was shown in Fig 4G-I, N=3-6 each group. All data were presented as mean ± SEM. One-way ANOVA test followed by Tukey’s post hoc test. *, *p* < 0.05, **, *p* < 0.01, ***, *p* < 0.001, ****, *p* <0.0001 vs P301S.


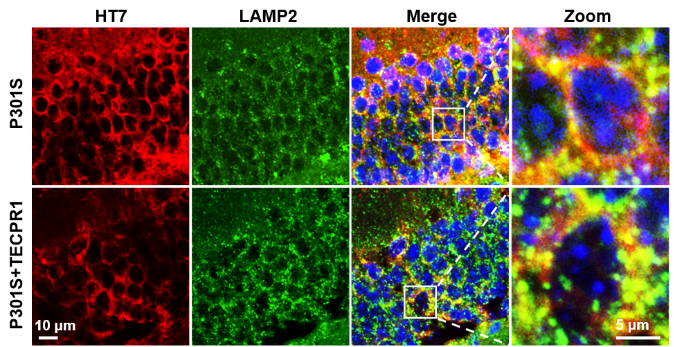


**Figure S7. Overexpression of TECPR1 promoted an increase colocalization of LAMP2 with HT7.** AAV-TECPR1-3×FLAG or AAV-3×FLAG virus was injected stereotaxically into the hippocampus of 8-month-old P301S-tau mice or the age-matched wild-type littermates, co-localization of HT7 and LAMP2 was measured by immunofluorescence.


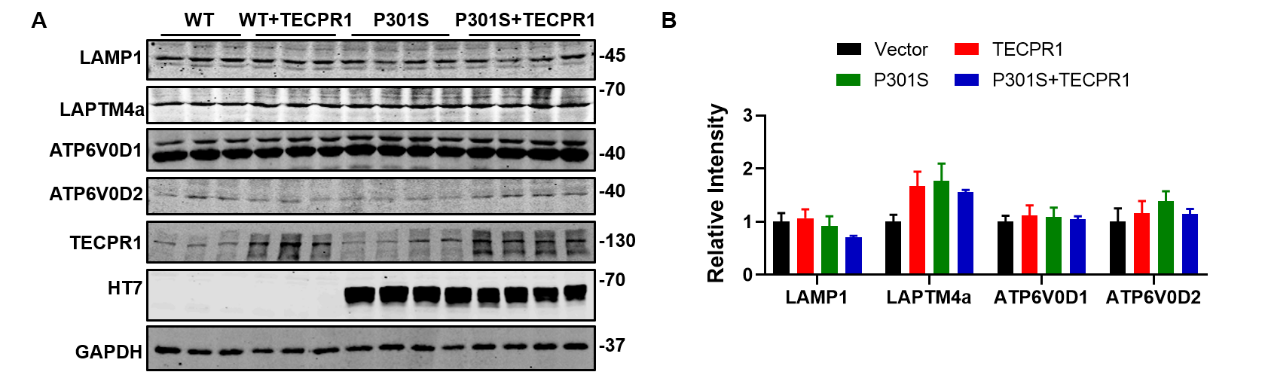


**Figure S8. TECPR1 did not alter the levels of lysosome-associated proteins.** **(A, B)** AAV-TECPR1-3×FLAG or AAV-3×FLAG virus was injected stereotaxically into the hippocampus of 8-month-old P301S-tau mice or the age-matched wild-type littermates. lysosome-associated proteins were detected by western blotting **(A)** and quantitative analysis **(B)**. N=3-4 each group. All data were presented as mean ± SEM. One-way ANOVA test followed by Tukey’s post hoc test for all data.


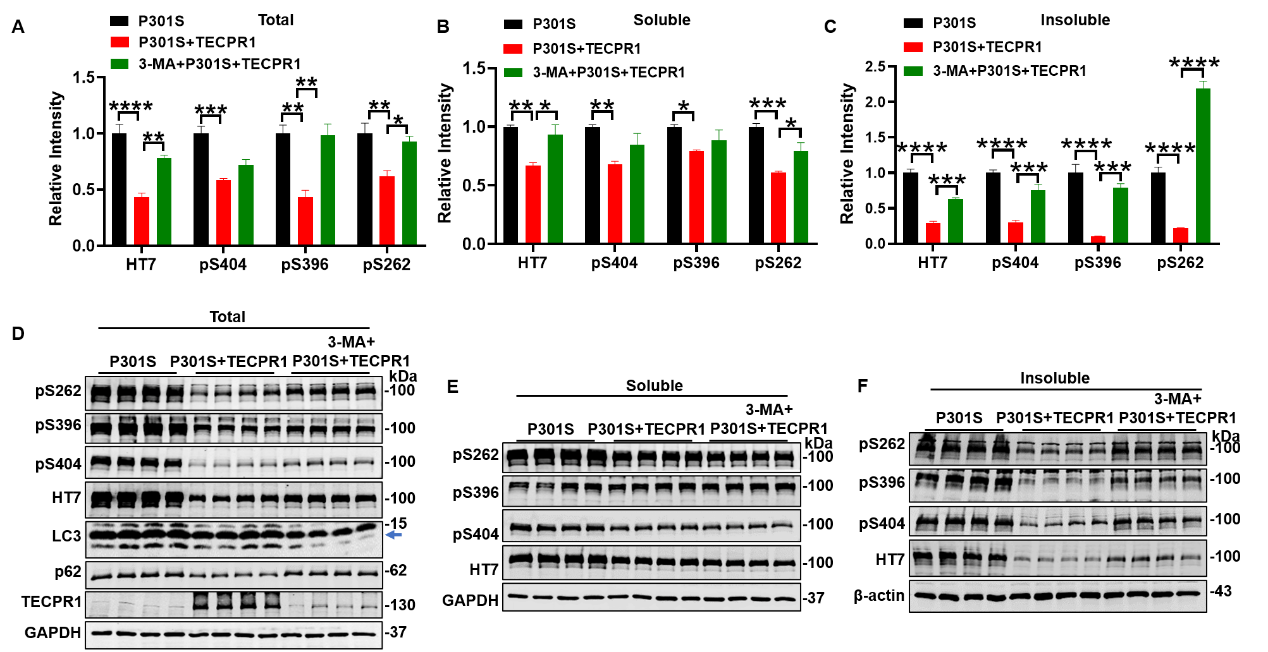


**Figure S9.** **3-MA inhibited the effect of TECPR1 reducing tau protein.** AAV-hSyn -TECPR1-3×FLAG virus was injected stereotaxically into the hippocampus of 8-month-old P301S-tau mice, followed by daily intraperitoneal injection of 3-MA (45 mg/kg) for 30 days. Quantitative analysis of total tau (HT7) and phosphorylated tau at Ser404, Ser396, Ser202 in total **(A)**, soluble **(B)** and insoluble fraction **(C)** of hippocampus, western blotting was shown in Fig 6I-K, N=4 each group. **(D-F)** Overexpression of P301S-tau and TECPR1 in HEK293 cells, followed by administration of 3-MA (5 mM) for 24 h, and total tau (HT7) and phosphorylated tau at Ser404, Ser396, Ser202 in total **(D)**, soluble **(E)** and insoluble **(F)** fraction were detected by western blotting. The blue arrow indicates LC3-II. N=4 each group. All data were presented as mean ± SEM. One-way ANOVA test followed by Tukey’s post hoc test. *, *p* < 0.05, **, *p* < 0.01, ***, *p* < 0.001, ****, *p* < 0.0001.


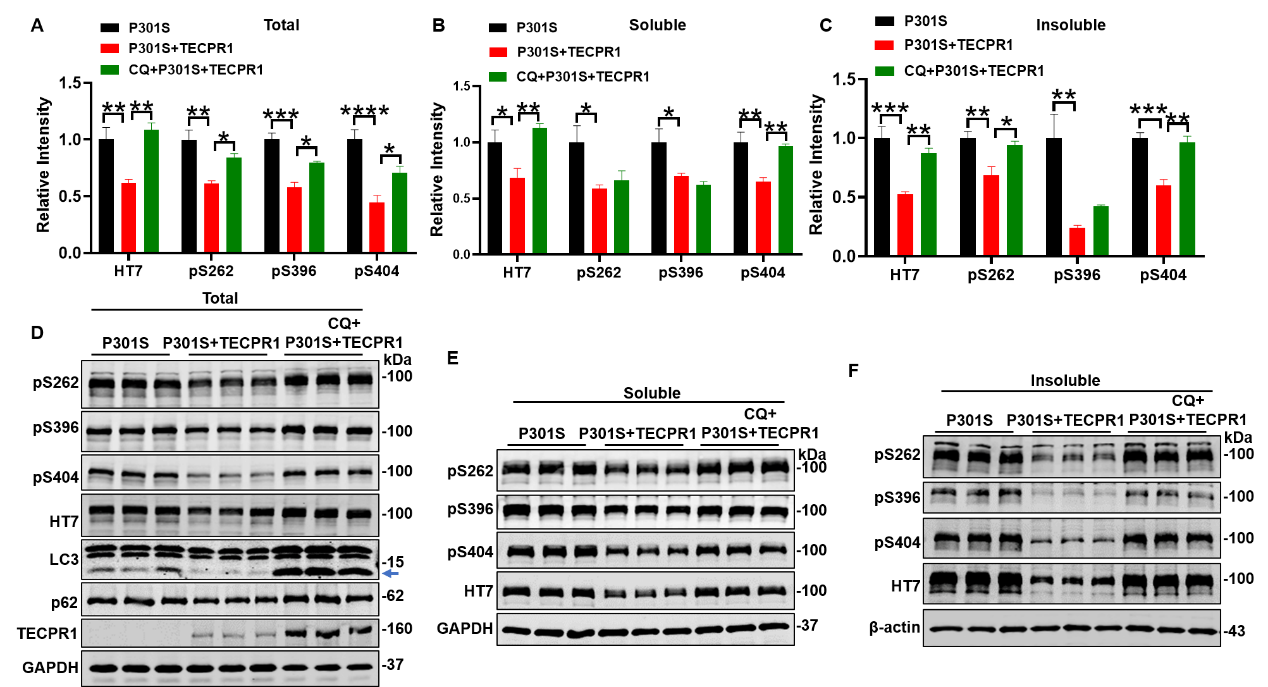


**Figure S10.** **CQ inhibited the effect of TECPR1 reducing tau protein.** AAV-hSyn-TECPR1-3×FLAG virus was injected stereotaxically into the hippocampus of 8-month-old P301S-tau mice for 15 days, followed by daily intraperitoneal injection of CQ (50 mg/kg) for 15 days. Quantitative analysis of total tau (HT7) and phosphorylated tau at Ser404, Ser396, Ser202 in total **(A)**, soluble **(B)** and insoluble fraction **(C)** of hippocampus, western blotting was shown in Fig 6T-V, N=4 each group. **(D-F)** Overexpression of P301S-tau and TECPR1 in cells, followed by administration of CQ (20 μM) for 24 h, and total tau (HT7) and phosphorylated tau at Ser404, Ser396, Ser202 in total **(D)**, soluble **(E)** and insoluble **(F)** fraction were detected by western blotting. The blue arrow indicates LC3-II. N=4 each group. All data were presented as mean ± SEM. One-way ANOVA test followed by Tukey’s post hoc test. *, *p* < 0.05, **, *p* < 0.01, ***, *p* < 0.001, ****, *p* < 0.0001.

**Supplementary Table S1. Antibodies used in the study**

| Antibody | Source | Identifier | Dilution |
| --- | --- | --- | --- |
| TECPR1 | Cell Signaling Technology | 8097S | WB:1/500 |
| TECPR1 | Thermo Fisher | PA5-54320 | IHC/IF:1/100 |
| GFAP | Cell Signaling Technology | 3670S | IHC/IF:1/500 |
| NeuN | Abcam | ab104224 | IHC/IF:1/500 |
| Iba1 | Abcam | ab5076 | IF:1/500 |
| HT7 | Thermo Fisher | MN1000 | WB:1/1000 |
| Tau5 | Abcam | ab80579 | WB:1/1000 |
| Phospho-tau（Ser396） | Signalway Antibody | 11102 | WB:1/1000 |
| Phospho-tau（Ser404） | Signalway Antibody | 11112 | WB:1/1000 |
| Phospho-tau（Ser262） | Signalway Antibody | 11111 | WB:1/1000 |
| PSD95 | ABclonal | A6194 | WB:1/1000 |
| PSD93 | Cell Signaling Technology | 19046S | WB:1/1000 |
| SYP | proteintech | 67864-1-Ig | WB:1/1000 |
| GluNR1 | ABclonal | A7167 | WB:1/1000 |
| SYT | ABclonal | A0992 | WB:1/1000 |
| LC3 | Abcam | ab51520 | WB:1/300 |
| LC3 | Abcam | ab48394 | IF:1/200；  WB:1/400 |
| p62 | ABclonal | A21703 | WB:1/1000 |
| LAMP2 | Abcam | Ab13524 | IF:1/300 |
| mTOR | proteintech | 66888-1-Ig | WB:1/1000 |
| p-mTOR（Ser2448） | proteintech | 67778-1-Ig | WB:1/1000 |
| p-p70S6K1（Thr389） | Cell Signaling Technology | 9205S | WB:1/500 |
| p70S6K1 | proteintech | 14485-1-AP | WB:1/300 |
| ATG13 | proteintech | 66708-1-Ig | WB:1/1000 |
| Beclin1 | proteintech | 66665-1-Ig | WB:1/1000 |
| p-ULK1（Ser757） | Cell Signaling Technology | 14202S | WB:1/500 |
| ULK1 | Cell Signaling Technology | 8054S | WB:1/300 |
| FLAG | CST | 14793S | IF:1/1000 |
| LAMP1 | Sigma | L1418 | WB:1/1000 |
| LAPTM4a | proteintech | 30627-1-AP | WB:1/200 |
| ATP6V0D1 | proteintech | 18274-1-AP | WB:1/500 |
| ATP6V0D2 | Sigma | SAB2103220 | WB:1/500 |
| GAPDH | proteintech | 60004-1-Ig | WB:1/2000 |
| DM1A | ABclonal | A7277 | WB:1/1000 |
| β-actin | ABclonal | AC038 | WB:1/2000 |
